# Mitochondria as Key Players in the Pathogenesis and Treatment of Rheumatoid Arthritis

**DOI:** 10.3389/fimmu.2021.673916

**Published:** 2021-04-29

**Authors:** Sally A. Clayton, Lucy MacDonald, Mariola Kurowska-Stolarska, Andrew R. Clark

**Affiliations:** ^1^ Institute of Inflammation and Ageing, University of Birmingham, Birmingham, United Kingdom; ^2^ Research into Inflammatory Arthritis Centre Versus Arthritis (RACE), Birmingham, United Kingdom; ^3^ Research into Inflammatory Arthritis Centre Versus Arthritis (RACE), Glasgow, United Kingdom; ^4^ Institute of Infection, Immunity, and Inflammation, University of Glasgow, Glasgow, United Kingdom

**Keywords:** mitochondria, rheumatoid arthritis, metabolism, oxidative phosphorylation, NLRP3, DAMP, DMARD (disease modifying anti-rheumatic drug)

## Abstract

Mitochondria are major energy-producing organelles that have central roles in cellular metabolism. They also act as important signalling hubs, and their dynamic regulation in response to stress signals helps to dictate the stress response of the cell. Rheumatoid arthritis is an inflammatory and autoimmune disease with high prevalence and complex aetiology. Mitochondrial activity affects differentiation, activation and survival of immune and non-immune cells that contribute to the pathogenesis of this disease. This review outlines what is known about the role of mitochondria in rheumatoid arthritis pathogenesis, and how current and future therapeutic strategies can function through modulation of mitochondrial activity. We also highlight areas of this topic that warrant further study. As producers of energy and of metabolites such as succinate and citrate, mitochondria help to shape the inflammatory phenotype of leukocytes during disease. Mitochondrial components can directly stimulate immune receptors by acting as damage-associated molecular patterns, which could represent an initiating factor for the development of sterile inflammation. Mitochondria are also an important source of intracellular reactive oxygen species, and facilitate the activation of the NLRP3 inflammasome, which produces cytokines linked to disease symptoms in rheumatoid arthritis. The fact that mitochondria contain their own genetic material renders them susceptible to mutation, which can propagate their dysfunction and immunostimulatory potential. Several drugs currently used for the treatment of rheumatoid arthritis regulate mitochondrial function either directly or indirectly. These actions contribute to their immunomodulatory functions, but can also lead to adverse effects. Metabolic and mitochondrial pathways are attractive targets for future anti-rheumatic drugs, however many questions still remain about the precise role of mitochondrial activity in different cell types in rheumatoid arthritis.

## Introduction

Mitochondria have long been described as central energy-producing organelles and regulators of cellular metabolism, but throughout the years many additional cellular functions of mitochondria have become apparent. It is now appreciated that these complex organelles also contribute to intra- and inter-cellular signalling through the actions of proteins, DNA, lipids, metabolites, and reactive oxygen species, and mitochondrial components are capable of directly activating the immune system.

Rheumatoid arthritis (RA) is a chronic inflammatory and autoimmune condition driven by a complex interplay of different immune and non-immune cell types. Dysregulation of immune signalling pathways causes local inflammation within the synovial joint, as well as a host of systemic complications such as an increased risk of cardiovascular diseases, all of which pose a significant risk to the affected individual’s quality of life (1). Known risk factors for the development of RA are both genetic and environmental, with the most important genetic link being the “shared epitope” of the MHC class II HLA-DR allele (1). Auto-antibodies such as rheumatoid factor or anti-citrullinated protein antibodies (ACPA) are found in a high percentage, though not all, of RA patients, and are associated with a more severe disease, but the presence of these antibodies generally pre-dates the development of clinical symptoms of arthritis by several years (2). It is considered that an assortment of cumulative triggering events, which are variable and still incompletely understood, then lead to the progression of a pre-clinical arthritis into established RA (1). Active RA disease involves expansion of the synovial membrane of the joint to form an invasive pannus, leading to joint damage and dysfunction. The identification of different subtypes of RA, which show varying degrees of contribution from infiltrating leukocytes to synovial pathology, highlights the heterogeneity of this disease and indicates that a range of diverse mechanisms likely drive disease pathogenesis in different individuals (1, 3, 4).

Metabolic dysregulation is a key contributing factor to the initiation and development of disease in autoimmunity, and much recent work has focused on the study of metabolic processes in RA and other inflammatory diseases in order to better understand and treat these complex conditions. Due to their pleiotropic effects on the cell, mitochondria contribute to disease pathogenesis *via* metabolic actions as well as through direct effects on signalling pathways.

In this review we address the contribution of mitochondria to pathological processes in RA, as well as how mitochondrial function can be altered by therapeutics in the context of RA treatment. We focus on the metabolic function of mitochondria in different cell types in RA, as well as how this organelle facilitates immune cell activation and production of inflammatory mediators. This has mostly been studied in T cells, macrophages, and fibroblasts, but there is also some evidence for mitochondrial regulation of endothelial cells, osteoclasts, neutrophils and chondrocytes in the context of arthritis. Mitochondria have a well-established role in the process of apoptosis, however this topic is outside the scope of the current review except where it directly relates to metabolic processes, and we direct the readers to other reviews on the topic (5), including in the context of rheumatoid arthritis (6, 7).

## Mitochondrial Dynamics

### Mitochondrial Fusion Versus Fission

The opposing processes of fusion and fission regulate the gross structure and overall organisation of mitochondria, and impact upon many aspects of mitochondrial function, including DNA segregation, oxidative phosphorylation efficiency, reactive oxygen species (ROS) production, and apoptosis. These mitochondrial activities can in turn impact cellular function in a multitude of ways, therefore mitochondrial dynamics plays a key role in cellular homeostasis and signalling (8). The balance of fusion and fission regulates the activity and survival of immune cells including T cells and macrophages. A fragmented mitochondrial state is observed in effector T cells, as well as macrophages treated with lipopolysaccharide (LPS) or infected with *Mycobacterium tuberculosis in vitro*, and this fission is linked to inflammatory cell function (8–11). In contrast, LPS treatment of human monocytes has been linked to a large, fused mitochondrial state (12, 13). Hyperfused mitochondria with tight cristae are observed in memory T cells, which retain high oxidative capacity and generate ATP through fatty acid oxidation and oxidative phosphorylation (9).

Due to their central role in mitochondrial regulation and cellular homeostasis, the fusion and fission processes are tightly controlled by a variety of interconnected mechanisms. In monocytes, the microRNA miR-125b regulates mitochondrial dynamics and apoptosis in several ways, including by downregulation of the mitochondrial fission protein MTP18 (12). Expression of miR-125b was found to be reduced in peripheral monocytes from RA patients compared with healthy individuals, although the precise contribution of this finding to disease processes has not been determined (12). T cells in systemic lupus erythematosus (SLE) contain large, fused mitochondria characterised by high membrane potential and excessive ROS production (14), however dysregulated fusion/fission processes have not been described in T cells from RA patients to our knowledge.

In addition to the regulation of immune cell function, mitochondrial dynamics also play a role in stromal cell activity. Fibroblasts are a major cell type present in the healthy synovial membrane, and these cells significantly contribute to disease processes in RA (15). Proliferation of fibroblasts leads to formation of the expanded synovial pannus, and these cells mediate extracellular matrix and cartilage degradation, contribute to immune cell recruitment and activation, and activate endothelial cells (15). Synovial tissue and *ex vivo*-cultured fibroblast-like synoviocytes (FLS) from RA patients show shortened mitochondria and elevated expression of the mitochondrial fission GTPase dynamin 1-like protein (DNM1L, also known as dynamin-related protein 1: Drp1) (16, 17). Inhibition of mitochondrial fission with the GTPase inhibitor m-divi in a collagen-induced arthritis (CIA) mouse model reduced disease severity, decreased synovial tissue ROS levels, and inhibited expression of inflammatory and destructive mediators (17).

Determining the precise contributions of fusion and fission processes to cellular function and disease is complicated by the fact that the machinery responsible for these processes also serve additional roles (8). For example, the fusion GTPase Opa1 also regulates mitochondrial cristae structure, which in turn modulates supercomplex formation and apoptosis (18); and the fusion-related protein mitofusin 2 has been linked to inflammasome activation and interleukin-1β (IL-1β) production, which shall be discussed in more detail in a later section (19). The specificity of tools that are commonly used to inhibit such machinery has also been brought into question, for example m-divi was shown to inhibit mitochondrial complex I activity independently of any effects on fusion/fission (20).

### Mitochondrial Biogenesis Versus Mitophagy

As well as showing dynamic regulation in the form of fusion and fission, cellular mitochondrial content is regulated by the balance of mitochondrial biogenesis and the degradation and recycling of mitochondrial components through autophagy – termed mitophagy. During homeostasis, this balance helps to maintain a healthy and functional population of mitochondria and may be indicative of overall respiratory capacity. The balance of fusion/fission and biogenesis/mitophagy are exquisitely sensitive to metabolic cues, such as nutrient availability and mitochondrial membrane potential (21). Cellular stress can be associated with an imbalance of these processes, and accumulation of damaged mitochondria can drive cellular dysfunction and/or immune cell activation. Disruptions to these pathways have been linked to a wide range of diseases, including cancers and several neurodegenerative diseases (21, 22).

The precise impact of mitophagy or its disruption on RA pathogenesis is poorly understood. Dysregulation of autophagy, whether increased or decreased, has been demonstrated in several different cell types in RA and has been linked to pathogenic processes (16, 23, 24), however the distinction between mitophagy and general autophagy in these studies has not been made. Clearance of defective mitochondria by mitophagy is important for the survival of chondrocytes from osteoarthritis (OA) patients, therefore this process likely has a role in protecting against cartilage loss in arthritis, but a specific link to rheumatoid arthritis was not shown (25).

Total mitochondrial mass is maintained in RA T cells, despite a decrease in respiratory activity in these cells compared with healthy controls (26). In contrast, T cells in SLE show higher mitochondrial load, attributable to increased biogenesis and decreased mitophagy (14). It is plausible that both mitochondrial biogenesis and mitophagy are disrupted in RA T cells, as it has been shown that activation of the energy sensing kinase AMPK is dysfunctional in these cells (27). AMPK serves as a crucial regulator of metabolic processes and acts to maintain mitochondrial homeostasis (28). The fact that AMPK is responsible for stimulating both mitophagy and mitochondrial biogenesis could explain the lack of changes in total mitochondrial mass in RA T cells. Activation of AMPK is also required to initiate mitochondrial fission processes in response to mitochondrial damage (28). The disrupted activity of AMPK in RA T cells results in uncontrolled activity of the mTOR complex mTORC1, which promotes T cell proliferation and inflammatory Th1 and Th17 differentiation that are associated with synovitis (27). mTOR is also a known regulator of mitochondrial dynamics through the control of mitochondrial protein synthesis (29). Whether the disruption of these energy sensing pathways in RA gives rise to a pool of dysfunctional mitochondria and contributes to oxidative stress needs to be determined.

## Mitochondrial Metabolic Activity and ATP Production

**Figure 1:**
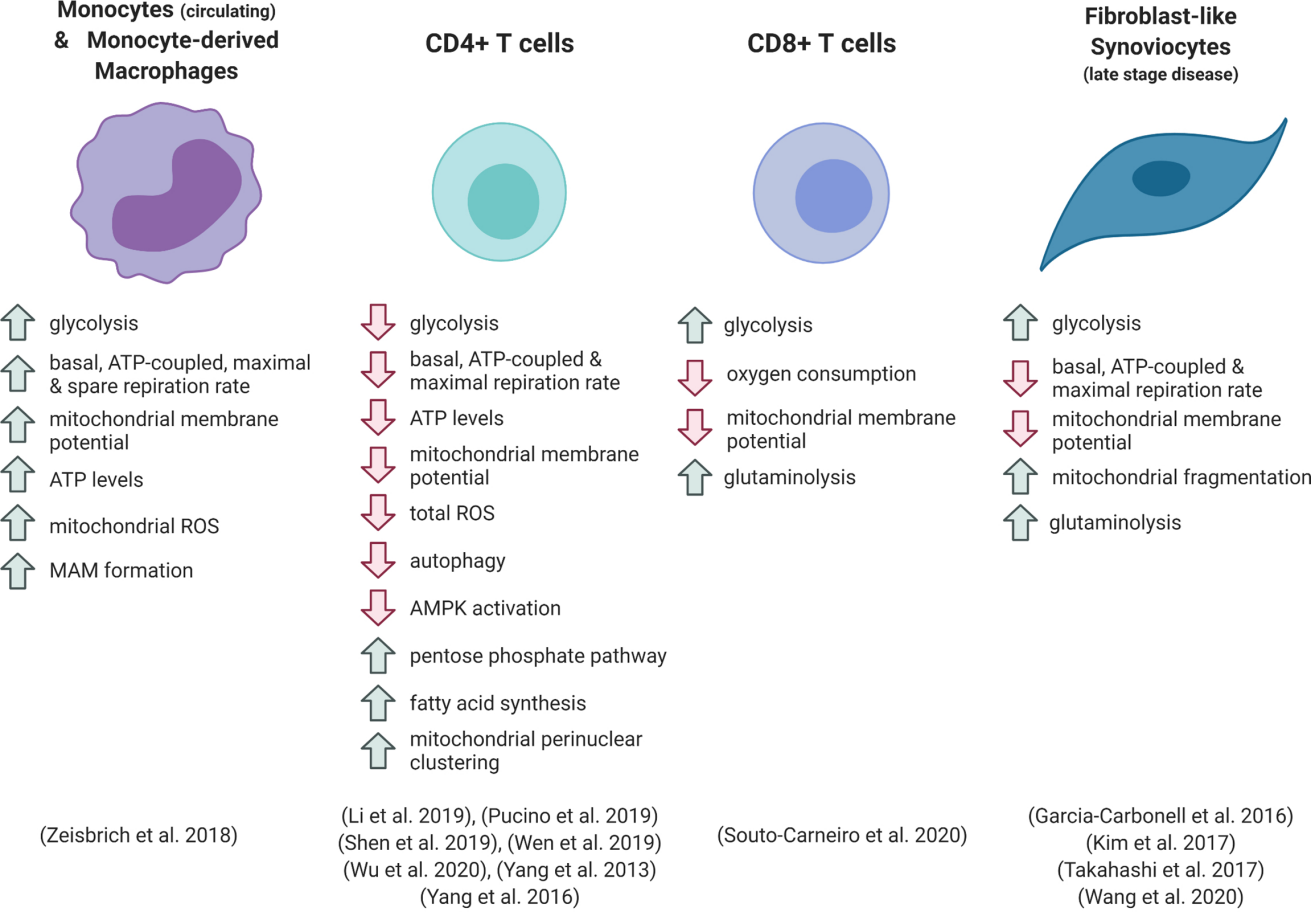
Metabolic phenotype of different cell types in rheumatoid arthritis. ROS, reactive oxygen species; MAM, mitochondria-associated membrane.

One of the central functions of the mitochondrion is the generation of adenosine triphosphate (ATP), which acts as the cell’s energy currency. ATP is produced in high quantities by the process of oxidative phosphorylation, carried out by the mitochondrial respiratory chain (aka electron transport chain/ETC) within the inner mitochondrial membrane. Coupling of electron transport and proton transfer produces an electrochemical gradient across the inner mitochondrial membrane (known as the mitochondrial membrane potential), allowing proton-driven phosphorylation of ADP by the enzyme ATP synthase (30). Mitochondrial respiration consumes oxygen, therefore cellular consumption of oxygen is commonly used as a readout of mitochondrial respiratory chain activity, with the ATP synthase inhibitor oligomycin being used to distinguish ATP-coupled oxygen consumption from other oxygen-consuming processes (30). The process of glycolysis involves the metabolism of glucose in the cytoplasm to generate pyruvate or lactate, allowing ATP production without oxygen consumption. Akin to the Warburg effect that is critical for cancer cell survival and proliferation, an increased rate of aerobic glycolysis is strongly linked to inflammatory activity in a number of different immune and stromal cell types (31, 32). Mitochondrial activity in inflammatory contexts, particularly in the context of inflammatory disease, is more varied and is still incompletely understood, however important advances in our understanding have been made in recent years (**Figure 1**).

### Monocytes and Synovial Tissue Macrophages

Inflammatory activation of macrophages, dendritic cells and monocytes results in upregulation of glycolytic metabolism, which is necessary for many of the pro-inflammatory functions of these cells (13, 32, 33). Numerous *in vitro* studies, which have predominantly focused on cells of mouse origin, show that this increase in glycolysis is accompanied by a robust downregulation of oxygen consumption and mitochondrial ATP production. This is thanks to the repurposing of the electron transport chain (ETC) and the tricarboxylic acid (TCA) cycle for ROS generation and the provision of specific metabolites for signalling and biosynthetic functions. These processes are reviewed extensively elsewhere (32–35), and are further discussed below. However, recent evidence has shown that metabolic responses to toll-like receptor 4 activation with LPS differ in human myeloid cells, and that different forms of inflammatory stimulus result in distinct metabolic phenotypes (13, 36). Therefore, the study of these pathways in the context of inflammation in human disease is required to fully appreciate the immunometabolic landscape of disease.

Circulating monocytes and monocyte-derived macrophages from the peripheral blood of RA patients are hyper-metabolic, displaying enhanced rates of both glycolysis and oxidative phosphorylation (37). Patient-derived cells showed elevated basal, ATP-linked and maximal oxygen consumption under *ex vivo* analysis compared with cells from healthy individuals (37). RA patient-derived macrophages had increased numbers of mitochondrial-ER contacts, forming structures known as mitochondria-associated membranes (MAMs). These structures facilitate calcium transfer between organelles, which can increase mitochondrial enzyme efficiency. The increased mitochondrial-ER associations were linked to deactivation of glycogen synthase kinase 3b (GSK3b), a kinase implicated in the regulation of mitochondrial respiratory activity. Increased levels of the inactive, phosphorylated form of GSK3b were detected in RA patient blood monocytes and synovial CD68+ macrophages. From a functional perspective, these metabolic adaptations were linked to increased macrophage production of the collagenase cathepsin K (37). This enzyme is involved in bone resorption and contributes to joint destruction in arthritis (38). Zeisbrich et al. demonstrated that the activity of cathepsin K correlated with RA disease activity (37). Cathepsin K is also associated with atherosclerotic lesions, and the authors suggest that the hypermetabolic and destructive phenotype of the RA patient macrophages, which mechanistically mirror that of coronary artery disease macrophages, may increase the risk of systemic complications associated with disease, such as cardiovascular complications (37). Whether this metabolic phenotype in the periphery is a cause or a consequence of joint inflammation is difficult to determine, and further investigation is required to understand whether macrophages in the synovium share this hypermetabolic signature.

**Figure 2:**
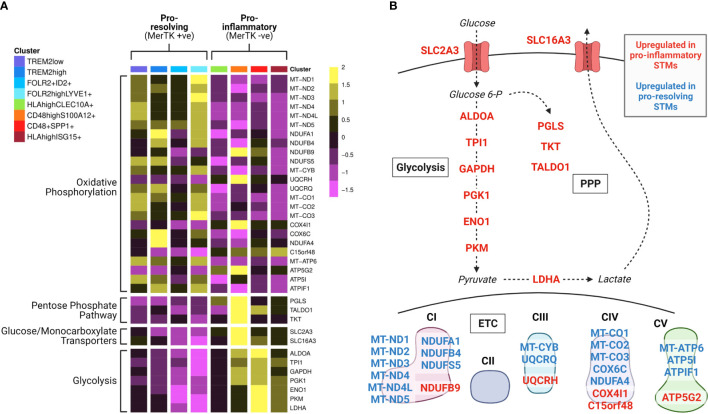
Synovial tissue macrophage subpopulations display distinct metabolic gene expression signatures. Data from Alivernini et al. (39). **(A)** Heatmap of synovial tissue macrophage single cell RNA sequencing data displaying differential expression of metabolism-associated genes across macrophage subpopulations (clusters). Detailed information on the characterisation and function of different clusters and their contribution to different disease outcome groups can be found in Alivernini et al. (39). **(B)** Pathway schematic of the data from **(A)**. Genes upregulated in at least one cluster of pro-inflammatory macrophages are displayed in red, and genes upregulated in at least one cluster of pro-resolving macrophages are displayed in blue. STM, synovial tissue macrophage; PPP, pentose phosphate pathway; ETC, electron transport chain.

Recent studies identified distinct types of synovial tissue macrophages with either protective or inflammatory functions in mouse and humans (39–41), and showed that these synovial tissue macrophage subpopulations appear to have opposing preferences for mitochondrial *versus* glycolytic metabolism (39). In the human synovium, macrophage subsets that express the TAM receptor MerTK predominantly display a pro-resolution gene expression signature and are associated with the healthy joint and RA disease remission (39). These subsets show elevated expression of genes linked to oxidative phosphorylation, including eleven out of the thirteen mitochondrially-encoded ETC subunit genes (**Figure 2**). In contrast, subsets lacking MerTK, which express high levels of pro-inflammatory mediators and induce an inflammatory and destructive phenotype in co-cultured fibroblasts, display elevated expression of genes of glycolysis, the pentose phosphate pathway (PPP) and transporters for glucose and lactate (**Figure 2**) (39). These expression profiles support our current understanding of general concepts of macrophage immunometabolism, that inflammatory macrophage function is driven by a skewing of metabolic activity towards glycolysis and the PPP, and away from mitochondrial ATP production (42). However, it remains to be seen precisely how the activity of different metabolic pathways varies amongst subpopulations of cells in RA, and to what extent these metabolic phenotypes dictate cellular function *in vivo*.

### T Cells

The metabolic regulation of T cells is crucial to allow their proliferation and effector functions following activation. Upon antigen recognition, quiescent naïve T cells transition into rapidly expanding effector T cells. These effector cells are driven by high rates of glycolysis, which allows fast energy production and provides substrates for DNA, lipid and protein synthesis. In contrast, long-lived memory T cells upregulate catabolic processes and depend upon mitochondrial oxidative metabolism (8). Inflammatory microenvironments such as the RA synovium induce metabolic reprogramming of T cells, which contributes to pathogenic processes. Retention of CD4+ T cells within the inflamed tissue is brought about by uptake of lactate, which drives increased fatty acid synthesis and elevated production of IL-17 (43).

In contrast to the hypermetabolic RA monocytes, circulating CD4+ T cells from RA patients show reductions in both oxygen consumption and lactate production in comparison with healthy controls. Consequently, ATP production rate and intracellular ATP levels are reduced in patient cells (24, 26, 44, 45). Several mechanisms have been linked to these phenomena.

One such mechanism is the diversion of glycolytic flux away from the mitochondria and lactate production, with carbon instead being rerouted into the PPP for production of NADPH and nucleotides. This was found to be achieved through the balance of two key rate-limiting enzymes, PFKFB3 and G6PD (44). PFKFB3 produces fructose 2,6-bisphosphate, the allosteric activator of the glycolytic enzyme PFK-1, and its upregulation in response to T cell activation was attenuated in RA T cells (24). In contrast, glucose-6-phosphate dehydrogenase (G6PD), which catalyses the first and rate-limiting step of the PPP, was over-expressed in RA T cells. This resulted in enhanced NADPH production and elevated levels of reduced glutathione in the patient cells. The increased antioxidant capacity was found to hinder the signalling function of intracellular ROS that are produced upon TCR activation, preventing activation of the cell cycle checkpoint kinase ATM, and giving rise to hyperproliferative T cells skewed towards inflammatory Th1 and Th17 differentiation (24, 44). Intracellular signalling by reactive oxygen species is also an important factor in other aspects of inflammatory cell function, which shall be discussed later.

In addition to reduced pyruvate entry into the mitochondria, RA CD4+ T cells possess a disrupted TCA cycle, which contributes to the loss of mitochondrial activity and reduced mitochondrial membrane potential in these cells (45). Specifically, T cells from RA patients have low activity of the mitochondrial enzyme succinyl-CoA ligase due to suppressed expression of the *SUCLG2* subunit gene. This was shown to result in elevated levels of upstream metabolites α-ketoglutarate, citrate and acetyl-CoA. From a functional perspective, this metabolic disruption increased acetylation of tubulin and promoted T cell motility and migration, resulting in greater pro-inflammatory potential and synovial invasiveness *in vivo* (45). Mitochondria were shown to cluster to a perinuclear region within RA T cells, which was also dependent upon tubulin acetylation, and which likely contributes to the regulation of mitochondrial signalling in these cells (45).

These findings demonstrate the intimate and inter-connected relationships between different metabolic pathways and their interplay with signalling pathways and multiple aspects of cellular functionality, as well as the relevance of these relationships to inflammatory disease.

Peripheral blood CD8+ T cells from RA patients demonstrate enhanced glycolysis and lactate production compared with healthy controls or other types of inflammatory arthritis (46). This glycolytic phenotype was accompanied by decreased mitochondrial membrane potential and oxygen consumption in RA patient cells. A glycolytic gene expression signature was also evident in CD8+ T cells from the RA synovium, which are predominantly present in ectopic lymphoid follicles. The reduced dependence on oxidative metabolism in the RA patient cells is thought to underlie their enhanced ability to proliferate in hypoxic conditions, and the RA T cells were shown to increase uptake of glutamine and its conversion to lactate when subjected to low oxygen and glucose (46). The increased lactate production from RA CD8+ T cells indicates that these cells are not subject to the same glycolytic checkpoints as seen in RA CD4+ T cells, however both metabolic phenotypes are associated with increased production of inflammatory mediators and are linked to disease processes.

### Fibroblast-Like Synoviocytes

Stromal cell metabolic function is also associated with RA pathogenesis. Fibroblasts consume glucose at high rates under inflammatory conditions, including in animal models of arthritis (47, 48). Fibroblast-like synoviocytes (FLS) from RA patients demonstrate a Warburg-like metabolic shift in comparison to OA FLS, favouring glycolysis over oxidative metabolism, and silencing of the glycolytic enzyme hexokinase-2 was able to reduce FLS migration and ameliorate disease in a serum-transfer arthritis model (47, 49). Kim et al. found that RA FLS show decreased basal respiratory rate and respiratory capacity compared with OA FLS, which was associated with mitochondrial depolarisation and abnormal morphology of cristae (16). These traits could be recapitulated *in vitro* by treatment of FLS with IL-17 or co-culture with Th17 cells, implicating inflammatory T cell activity in RA fibroblast dysregulation. Expression of several components of the mitochondrial ETC was decreased by IL-17 treatment (16). A hallmark of FLS in RA is their resistance to apoptotic signals, which contributes to FLS activation, synovial hyperplasia and formation of the invasive pannus (6). One mechanism that has been implicated in this apoptosis resistance is increased autophagy, which allows the cell to withstand nutrient deprivation and inhibits the ER stress response (50, 51). In the study by Kim et al., IL-17 treatment reduced apoptosis of RA FLS, and inhibition of autophagosome formation could reverse this effect. As mitochondrial dysfunction can be a trigger of autophagic processes, these results suggest a link between IL-17-mediated mitochondrial stress, autophagy and FLS survival, which could be an important pathway in disease progression (16).

Similarly to macrophages, recent studies have highlighted important distinctions between the roles of different subpopulations of T cells and fibroblasts in RA (52, 53). It will therefore be interesting to determine whether these different subsets also show distinct metabolic phenotypes that determine their specific functions.

### Other Cell Types

There is little study of mitochondrial activity in other immune cell types in RA, with the majority of the focus in this context being on macrophages and T cells. B cells contribute to various stages of RA pathogenesis through the production of autoantibodies and by contributing to cytokine production and antigen presentation (1). Activated B cells upregulate both glycolytic and oxidative metabolism to meet the high energetic demands of proliferation and antibody production, and mitochondrial mass increases upon B cell stimulation *in vitro* (54). Mitochondrial signalling, in particular mitochondrial ROS generation, is important for dictating specific B cell differentiation paths (55). The precise metabolic state of B cells in RA has not been described, however deletion of one allele of the *PPARγ* gene resulted in hyperproliferative and hyperresponsive B cells and exacerbated disease in an antigen-induced arthritis model. This suggests that lipid metabolism may be important in regulating B cell responses in RA (56).

Neutrophils also play a role in RA disease, contributing to inflammatory and destructive processes and acting as sources of autoantigens (57). Neutrophils rely heavily on glycolysis for energy production (58), and neutrophils from RA synovial fluid displayed an enhanced glycolytic gene signature compared with peripheral blood neutrophils from the same patients (57). Changes in mitochondrial activity have been shown to impact upon neutrophil functions such as chemotaxis (58), however this has not yet been demonstrated in the context of RA. Neutrophils are thought to be a source of immunostimulatory extracellular mitochondrial DNA in autoimmune diseases (59–61), which shall be discussed in a later section, but the metabolic regulation of neutrophils in RA warrants further study.

### Hypoxia

The precise contribution of mitochondrial activity to ATP production within the synovial environment is yet to be fully understood. One important factor to take into consideration is the availability of oxygen within this microenvironment, as the inflamed synovium has been demonstrated to be profoundly hypoxic. Synovial tissue oxygen tension (pO_2_) varies considerably between patients, but average measurements of around 20mmHg have been reported (equivalent to roughly 3% ambient concentration), and values as low as 3.2mmHg (0.45%) have been detected (62–64). *In vivo* pO_2_ was shown to negatively correlate with macroscopic synovitis, as well as with the numbers of CD3+ and CD68+ cells within the synovial sublining layer (64). The hypoxic microenvironment can exacerbate disease *via* many different mechanisms, which are reviewed in detail by McGarry et al. and Deng et al. (65, 66). These include the induction of oxidative stress, as well as the stabilisation of HIF-1α, which directly stimulates the production of inflammatory cytokines such as IL-1β and promotes glycolytic metabolism (65, 66).

A number of regulatory mechanisms (in the most part coordinated by HIF-1α) act to reduce mitochondrial activity during low oxygen conditions. This serves to prevent excessive ROS production at complexes I and III of the ETC (67). These mechanisms include decreasing expression and promoting subunit remodelling of ETC complexes, as well as reducing substrate entry into the mitochondrial TCA cycle through inhibition of pyruvate dehydrogenase (67–69). Hypoxia has also more recently been shown to cause reduced expression of the mitochondrial pyruvate carrier (MPC), which is critical for transport of glycolysis-derived pyruvate into the mitochondria (70). Chronic hypoxia may result in an additional suite of adaptations over those of an acute hypoxia insult. Exposure of the monocyte cell line THP1 to chronic hypoxia (72h) substantially reduced oxygen consumption compared with normoxia or acute hypoxia, however a low level of oxidative respiration was maintained through increased electron-transferring flavoprotein expression and oxidation of fatty acids and glutamine (71).

Hypoxia can also have cell type-specific effects. In contrast to other cell types, exposure of osteoclasts to hypoxia did not cause inhibition of pyruvate dehydrogenase activity, and these cells actually increased their mitochondrial activity in hypoxia compared with normoxia (72). This enabled increased ATP production, which is necessary for the increase in bone resorptive activity demonstrated by osteoclasts in hypoxia, suggesting that increased mitochondrial activity in osteoclasts may contribute to bone loss in severe RA (72).

The activity of mitochondria within synovial cell populations is therefore dependent upon the extent to which these regulatory mechanisms are deployed, and this highlights the importance of studying these cells within physiologically relevant conditions.

### Nitric Oxide

Another important factor affecting the activity of mitochondrial metabolism in immune cells is the production of nitric oxide (NO). This short-lived signalling molecule is produced from arginine by the nitric oxide synthase (NOS) enzymes, and inhibits mitochondrial metabolism through a variety of mechanisms. NO reversibly inhibits cytochrome *c* oxidase activity through competition for the oxygen binding site (73). In addition, NO irreversibly inhibits several mitochondrial enzymes through direct modification of cysteine residues by the process of nitrosylation. This particularly affects iron-sulfur cluster-containing enzymes, including the TCA cycle enzyme aconitase and complexes I and II of the ETC (73–75). NO signalling has also been linked to reduced abundance of complex I subunits, including key catalytic subunits (76). These mechanisms cooperatively lead to decreased mitochondrial activity and oxygen consumption.

The expression of inducible nitric oxide synthase (iNOS, encoded by the *Nos2* gene) and production of NO is a hallmark of mouse “M1”-type pro-inflammatory macrophages and LPS-treated dendritic cells. In these cells NO is implicated in the strong inhibition of oxidative metabolism following toll-like receptor activation, and contributes to cellular commitment to glycolysis (75–78). Despite this striking effect in mouse cells, human macrophages show little or no synthesis of NO following the same activation signals *in vitro*, which has been explained by the detection of extensive CpG methylation of the *Nos2* promoter in human cells (79–81). This likely underlies the observation that cultured human monocyte-derived macrophages do not downregulate mitochondrial oxygen consumption in response to LPS treatment (36). However, it has been shown that PBMCs from RA patients demonstrate detectable iNOS expression and produce NO (82), suggesting that this signalling molecule may be relevant to mitochondrial regulation in the inflammatory environment *in vivo*. These findings further stress the importance of studying metabolic processes under disease-relevant conditions, as well as the vital consideration of the differences between human disease and animal models (81).

Nitric oxide is also produced by T cells, and increased NO production by these cells in SLE has been linked to increased mitochondrial mass and mitochondrial hyperpolarisation (83). Elevated NO production was also detected in circulating T cells from RA patients compared with controls, although no differences in mitochondrial mass were detected between these groups (84). NO has also been suggested to influence T cell differentiation, however opposing effects have been reported on Th17 differentiation in different studies (79). Differing consequences of T cell-intrinsic production *versus* exogenous exposure, as well as opposing effects of large *versus* small quantities of NO on T cell survival, also complicate the formation of conclusions on the contribution of this signalling molecule to RA disease (79). However, the striking impact of NO on a variety of different metabolic processes means that this small molecule deserves further study in disease-relevant situations.

## Fatty Acid Oxidation

Oxidative phosphorylation is fuelled from several different sources. In addition to pyruvate derived from glycolysis and glutamate produced by the metabolism of glutamine, the TCA cycle can receive carbon from fatty acids through acetyl-CoA generation by the process of fatty acid β-oxidation (FAO). This process also generates NADH and FADH_2_, which can directly drive the mitochondrial respiratory chain. β-oxidation occurs within the mitochondria through a series of enzymatic reactions. The rate limiting step is entry of fatty acid acyl-CoA into the mitochondria, which involves conjugation to carnitine by the enzyme carnitine palmitoyltransferase I (CPT1) (32). FAO is an important mechanism of energy production in certain immune cell types, including regulatory T cells and “M2”-type macrophages (32). While FAO was previously described as essential for macrophage differentiation in response to IL-4 (M2 differentiation), as well as for memory T cell differentiation, the dependence of these cells on FAO has more recently been brought into question. This was thanks to evidence of non-specific effects of the inhibitor etomoxir, which is used to inhibit CPT1 activity (85, 86), as well as the finding that inhibition of FAO in human monocyte-derived macrophages did not prevent M2 gene expression or anti-inflammatory function (87). Despite these question marks, fatty acid metabolism has been shown to be disrupted in RA, although more work is required to fully understand the dysregulation of FAO processes in different cell types.

Rodgers et al. described a link between carnitine shuttling of fatty acids and the production of the chemokine CCL20, which has roles in lymphocyte recruitment and osteoclast activity (88). Treatment of human monocytes with exogenous carnitine enhanced LPS-induced CCL20 production, and culture of monocytes in RA synovial fluid led to an increase in intracellular carnitine metabolites under hypoxic conditions. It was proposed that entry of monocytes into the hypoxic and inflamed synovial joint brings about alterations in fatty acid dynamics, supporting the production of CCL20 and promoting further inflammation and joint damage (88). A different study found that carnitine was elevated in synovial fluid samples from RA patients compared with healthy subjects (89). However, this study also showed that enzymes involved in FAO, including HADHA and ACADVL, were significantly downregulated in RA FLS compared with healthy subjects (89). These results suggest that modulation of fatty acid metabolism may vary between different cell types in RA. Hypoxia may be a key regulator of these processes in the synovium, as silencing of HIF-1α in FLS could increase expression of FAO enzymes (89).

Fatty acid metabolism is also disrupted in T cells during RA disease. RA patient T cells accumulate lipid droplets within their cytoplasm through high rates of fatty acid synthesis, which facilitates T cell hypermotility and tissue invasion (90). Enzymes of the FAO pathway were found to be elevated in RA T cells, but there were no differences in expression of *Cpt1*. Therefore it remains to be seen how delivery of fatty acids into the mitochondria and their degradation are regulated in this context, and whether insufficient FAO contributes to the lipid accumulation and low levels of ATP seen in the patient T cells (90). It was subsequently shown that AMPK activation is dysfunctional in RA T cells (27), and AMPK is known to inhibit fatty acid synthesis and to activate mitochondrial fatty acid uptake (28). Therefore dysregulated energy sensing in these cells may result in an imbalance between β-oxidation and fatty acid synthesis, contributing to pathogenic activity of inflammatory T cells.

Elevated levels of free fatty acids of multiple types have been found in the synovial fluid of RA patients relative to healthy individuals (91), however fatty acids were lower in RA synovial fluid compared with other inflammatory arthritis types (92). Different types of fatty acid can have a range of pro- or anti-inflammatory functions *via* multiple mechanisms, including by directly stimulating immune cell receptors, or by influencing membrane synthesis and composition (93–95). Therefore, it is difficult to separate the energy-generating functions of fatty acids in the mitochondria from other roles in RA based solely on abundance measurements. Changes in fatty acid metabolism may also perpetuate extra-articular symptoms of RA, for example differential expression of enzymes involved in fatty acid metabolism were observed in skeletal muscle tissue in an arthritis model and RA patients (96).

## Glutaminolysis

The metabolism of amino acids has emerged as a vital process that drives both the proliferation of cancer cells and the function of immune cells, and which exceeds the requirement for protein synthesis (97, 98). The amino acid glutamine acts as the principal nitrogen donor for production of nucleic acids and non-essential amino acids, as well as contributing carbons to the mitochondrial TCA cycle through its conversion first to glutamate and subsequently to α-ketoglutarate *via* a process known as glutaminolysis (98). In this way glutamine metabolism is an important anaplerotic mechanism that helps to maintain TCA cycle flux (97–99). Glutaminolysis also contributes to epigenetic regulation through the generation of cofactors or inhibitors of chromatin remodelling enzymes (100–103), and affects protein modification through hexosamine biosynthesis (104).

The mitochondrial enzyme glutaminase 1 (GLS1) catalyses the first step of glutaminolysis: the deamidation of glutamine to glutamate. Takahashi et al. showed that expression of GLS1 was higher in RA FLS compared with those from OA patients, and glutamine starvation or knock-down of GLS1 significantly inhibited RA FLS proliferation. Pharmacological inhibition of glutaminase also reduced RA FLS proliferation both *in vitro* and *in vivo*, and significantly ameliorated disease in an SKG mouse model of arthritis (105). These results suggest that RA FLS exhibit a “glutamine addiction” similar to certain cancer cells (98, 105). However, this reliance on glutamine may relate only to specific pathological properties of FLS, such as proliferation, as inhibition of GLS1 did not alter FLS production of either IL-6 or matrix metalloproteinase-3 (105).

Activated T cells strongly upregulate both glucose and glutamine uptake, and through its numerous functional roles within the cell glutamine metabolism differentially impacts effector activity of different T cell subsets (101). The presence of glutamine has been shown to control the balance of differentiation between Treg cells and Th1 or Th17 cells (103, 106). Recent work reported that Th17 cells are more reliant upon glutaminolysis than are other T helper subsets, and GLS1 is preferentially upregulated in Th17 cells (101, 103, 107). GLS1 inhibition reduced Th17 proliferation and IL-17 production *in vitro*, and ameliorated Th17-driven inflammation *in vivo* in models of inflammatory bowel disease, allergic airway disease and experimental autoimmune encephalomyelitis (101, 107). Despite these reports of a dependence on glutaminolysis for Th17 cell differentiation and proliferation, Takahashi et al. found no difference in Th17 numbers in the spleen of SKG mice following administration of a GLS1 inhibitor (105). However in a different study, the glutamine antagonist 6-diazo-5-oxo-L-norleucine (DON) reduced the proportion of splenic Th17 cells, and showed an additive beneficial effect on arthritis severity when used along with the mTOR inhibitor rapamycin in the SKG mouse model (108). The precise role of glutaminolysis in T helper cell function in RA therefore requires further study.

As mentioned above, CD8+ T cells from RA patients were able to metabolise glutamine to lactate when subjected to low glucose conditions (46). Therefore, glutamine may be particularly important in the inflamed synovial environment, where competition for glucose is fierce due to the abundance of activated immune and stromal cells. RA CD8+ T cells strongly upregulated expression of the glutamine transporter *SLC5A1* upon stimulation, resulting in significantly elevated expression compared with healthy control cells (46).

Glutamine metabolism has also been shown to be important for monocyte/macrophage function. Glutamine feeds the TCA cycle in both LPS- and IL-4-treated macrophages (97, 99, 102, 104), and the abundance of glutamine-derived metabolites α-ketoglutarate, succinate and fumarate influences macrophage polarisation and innate immune memory through epigenetic mechanisms and prolyl hydroxylase regulation (99, 100, 102). How these mechanisms influence cellular activity in RA is thus far unknown.

A metabolomic study comparing different inflammatory arthropathies found that synovial fluid glutamine levels were highly elevated in RA patients in comparison with ankylosing spondylitis, Behçet’s disease or gout (92). Baseline levels of glutamine in the urine or serum of RA patients also contributed to two independent metabolite profiles that could distinguish clinical responders *versus* non-responders to anti-TNFα therapy, with elevated glutamine levels associating with a favourable response (109, 110). Priori et al. showed that serum glutamine levels significantly increased following six months of etanercept treatment in good responders (110). While it is difficult to directly infer mechanistic changes based on these metabolomic studies, the differences in glutamine levels may help to stratify patients for effective treatment strategies.

## TCA Cycle Metabolites

The tricarboxylic acid (TCA) cycle is a series of enzymatic reactions that occur in the mitochondrial matrix to produce reducing equivalents in the form of NADH and FADH_2_ to fuel the ETC. TCA cycle metabolites also participate in branching pathways for the biosynthesis of alternative small molecules and macromolecules, or can have signalling roles in their own rights (30). All of these functions have the potential to contribute to disease processes in inflammatory conditions such as RA. Measurement of metabolite concentrations in different biofluid samples from patients can give insights into mechanisms of disease, as well as identifying potential disease biomarkers that will assist in accurate diagnosis or prediction of response to therapy.

### Succinate

Succinate is produced from succinyl-CoA in the TCA cycle, and is metabolised to fumarate by the enzyme succinate dehydrogenase, which forms complex II of the ETC. Extracellular succinate was found to be elevated in the synovial fluid in an antigen-induced arthritis model compared with naïve mice, and the level of paw swelling correlated with the level of succinate in the synovial fluid (111). Succinate can also be found in the synovial fluid of patients with RA (111). Similarly to glutamine, succinate was strongly elevated in synovial fluid from RA patients compared with other forms of inflammatory arthritis (92). However, while succinate was detected in the synovial fluid in a separate comparison of several inflammatory and non-inflammatory arthritis types, no metabolic distinction was found between patient groups in this study (112). Metabolomic studies of plasma and serum found no significant differences in succinate levels between RA patients and controls (91, 113–115), or between RA patient groups with differing disease activity (114), meaning that succinate levels are unlikely to be a useful clinical biomarker of disease. It is unclear whether these different results represent mechanistic differences in disease processes between the circulation and synovium, or if the power of the studies was insufficient to detect differences in this metabolite in serum samples.

Macrophages are known to accumulate succinate following an inflammatory challenge, due to a break in the TCA cycle caused by inhibition of succinate dehydrogenase, as well as through increased anaplerosis of TCA cycle metabolites from glutamine and the GABA shunt (99, 116, 117). In addition to intracellular accumulation, macrophages have been shown to release succinate into the extracellular environment following LPS treatment *in vitro* (111). LPS treatment of endothelial cells also induces succinate accumulation, as does hypoxia exposure of both endothelial cells and synovial fibroblasts, indicating that several cell populations could contribute to elevated succinate levels within the synovial environment (118).

From a mechanistic perspective, succinate can have inflammatory effects in both the intra- and extra-cellular compartments. Accumulation of intracellular succinate in macrophages inhibits prolyl hydroxylase (PHD) enzymes either directly or through increased ROS generation (117). This leads to stabilisation of HIF-1α, promoting glycolytic metabolism and IL-1β production, amongst other effects (99). Inhibition of PHDs can also induce NF-κB-mediated inflammatory gene expression through increased activity of the kinase IKKβ, which promotes the degradation of IκBα, the negative regulator of NF-κB (102, 119).

Extracellular succinate can act as an alarmin or danger signal, enhancing immune cell activation by autocrine and paracrine signalling through the plasma membrane succinate receptor SUCNR1/GPR91 (120). This G protein-coupled receptor is expressed by several different cell types, including dendritic cells (DCs) and macrophages, where its expression is further enhanced following inflammatory stimulation (111, 120). Activation of SUCNR1 on macrophages enhances HIF-1α protein expression and augments production of IL-1β in response to inflammatory challenge. Deletion of the *Sucnr1* gene led to a reduction in synovial IL-1β levels and significantly reduced knee swelling in an antigen-induced arthritis model (111). Activation of SUCNR1 on DCs promotes their migration to lymph nodes and enhances costimulatory capacity of DCs towards T cells (120). This was linked to exacerbation of disease in antigen-induced arthritis through increased expansion of Th17 cells (121).

Succinate also promotes angiogenesis within the synovial membrane by stimulating the production of vascular endothelial growth factor (VEGF) by endothelial cells. This has been linked to intracellular accumulation of succinate in these cells leading to stabilisation of HIF-1α, as well as activation of endothelial cell SUCNR1 by extracellular succinate, both of which promote VEGF production (118). Endothelial cell activation and increased angiogenesis are known to contribute to pathogenic processes in RA, for example by enabling and promoting leukocyte recruitment and migration, as well as supporting hyperplasia of the synovial pannus (122). Treatment of RA synovial fibroblasts with succinate *in vitro* increased production of basic fibroblast growth factor (bFGF) and cellular invasion, which could also contribute to the invasive pannus during disease (123).

Despite these numerous reports of an inflammatory role for succinate signalling, several groups have instead described an anti-inflammatory function of this metabolite, including in isolated bone marrow-derived or peritoneal macrophages (124, 125), and in adipose tissue under steady state and in the context of obesity (125). These anti-inflammatory effects are suggested to occur through both SUCNR1-dependent and -independent mechanisms (124). Keiran et al. also reported that macrophage expression of *Sucnr1* is reduced by LPS treatment and increased by the type 2 cytokine IL-4 (125), which is in opposition to previously reported findings (111). In the context of cancer, tumour-derived succinate induced migration and IL-6 production in tumour-associated macrophages, but also increased *Arg1* expression, a marker of both “M2”-type and tumour-associated macrophages (126). Collectively these results suggest that cell type and context are key for the precise role of succinate in immune cell regulation.

The mechanism of export of succinate from cells of the synovium is also not fully understood. Release of succinate into the extracellular environment upon cell death and rupture is well documented and is consistent with the action of this metabolite as an alarmin (120). Membrane transport of succinate in viable cells is less well understood, although cancer cells have been shown to take up succinate in order to fuel mitochondrial metabolism *via* sodium-coupled dicarboxylic acid transporters such as NaDC3 (*SLC13A3*) (127). Reddy et al. also showed that the monocarboxylate transporter MCT1 (*SLC16A1*) can export succinate from active muscle cells, with important paracrine effects (128). Both of these transport activities required a low pH environment (127, 128). It remains to be seen whether these mechanisms are responsible for succinate transport in the synovial environment, which, similarly to active muscle and the tumour microenvironment, can exhibit localised acidic pH due to high glycolytic rates and enhanced lactic acid production (129, 130). MCT1 has also been shown to be expressed in the arthritic synovial joint (131).

### Citrate and Itaconate

Citrate is a TCA cycle metabolite produced from acetyl-CoA and oxaloacetate by the enzyme citrate synthase. Similarly to succinate, citrate accumulates in inflammatory macrophages due to interruption of the TCA cycle. Mechanisms implicated in this accumulation are downregulation of the downstream enzyme isocitrate dehydrogenase (IDH) (104), as well as NO-mediated inhibition of aconitase, the enzyme that converts citrate to isocitrate (75). Citrate also accumulates in RA T cells due to succinyl-CoA ligase deficiency and reversal of the TCA cycle, as well as through uptake and metabolism of lactate from the inflamed microenvironment (43, 45). Citrate is a key biosynthetic metabolite that supports inflammatory macrophage and T cell function. It is used to generate acetyl-CoA, which itself is utilised for the synthesis of fatty acids and lipids, including prostaglandins, and for protein modification. Citrate is also used for the production of NADPH, which is required for NO and ROS generation and to support antioxidant processes (42).

Another fate of intracellular citrate is its conversion into the immune-related metabolite itaconate by the enzyme aconitate decarboxylase 1 (ACOD1), also known as immune-responsive gene 1 protein (IRG1). *Irg1* is one of the most strongly induced genes upon LPS stimulation of macrophages, and intracellular itaconate accumulates to high levels (132). The original described function of itaconate was its direct anti-microbial action through inhibition of the glyoxylate shunt, but it has since been shown to have a number of immunomodulatory functions. These include but are not limited to: anti-inflammatory action through the inhibition of succinate dehydrogenase activity; antioxidant roles through indirect activation of the transcription factor Nrf2; and contribution to innate immune tolerance (116, 132–135).

Despite the accumulation of citrate in inflammatory macrophages *in vitro* and peripheral blood T cells from RA patients, levels of citrate were found to be significantly reduced in synovial fluid, serum and urine samples from RA patients in comparison with healthy individuals (89, 136). The decrease in synovial fluid citrate was linked to decreased expression of citrate synthase in RA patient synovial tissue. Other enzymes of the TCA cycle were also found to be reduced in RA synovial tissue in this study, including malate dehydrogenase and a component of the α-ketoglutarate dehydrogenase complex (DLST), suggesting an overall decrease in TCA cycle activity during disease (89).

An alternative explanation for the decrease in citrate levels in RA could be increased consumption of this metabolite in alternative pathways, for example for the production of itaconate, which has been detected in the plasma of patients with early RA (137). In these patients the change in itaconate levels showed highly significant negative correlation with changes in disease activity following the initial 3 months of conventional disease-modifying anti-rheumatic drug (DMARD) therapy, with a decrease in overall disease activity (DAS44) or measures of inflammation (CRP and ESR) being associated with an increase in plasma itaconate (137). While itaconate production is strongly induced by inflammatory stimuli, its anti-inflammatory and antioxidant properties could account for the negative correlation found here, and the role of this metabolite in the resolution of disease warrants further investigation.

In contrast with these findings in patients, a metabolomic analysis of the transgenic human TNFα mouse model of polyarthritis (Tg197) found that itaconate was high in samples from these transgenic mice following spontaneous disease development, whereas no itaconate was detected in wild-type mice and transgenic animals following treatment with infliximab (anti-hTNFα). The expression of the *Irg1* gene was accordingly higher in the hind limb tissue from transgenic animals relative to wild-type (138). This study also found higher levels of citrate in synovial fibroblasts of the transgenic mice compared with wild-type, in opposition to the decreased citrate seen in patient studies (136, 138). The differences in these studies may represent different disease mechanisms, as well as distinct mechanisms of action of conventional DMARDs and the anti-TNF biologic. These results also highlight the difficulties in interpretation of correlation data when it comes to deciphering mechanisms of disease or therapeutic activity, especially when it comes to feedback mechanisms such as anti-inflammatory mediators that are regulated by inflammatory signals.

The intracellular functions of itaconate are orchestrated both inside the mitochondrion (e.g. inhibition of succinate dehydrogenase) and in the cytosol (e.g. alkylation of KEAP1 to activate Nrf2), and itaconate can be transported across the mitochondrial membrane by the human citrate carrier and 2-oxoglutarate/malate carrier (135). Transporters of itaconate have been identified in certain fungal species that are utilised for industrial synthesis of itaconate (139), however the mechanism by which mammalian cells may excrete or take up itaconate is not known. As a dicarboxylic acid salt, itaconate is unable to freely cross the plasma membrane, and this has resulted in the use of various modified membrane-permeable forms of the metabolite to investigate its role in immune regulation (140). Puchalska et al. showed that exogenous unmodified itaconate could be taken up by bone marrow-derived macrophages, resulting in an altered metabolic fate of glucose. This occurred to a greater extent in unstimulated and IL-4-treated macrophages compared with LPS-treated, suggesting that uptake may be dependent upon an itaconate concentration gradient (141).

The extent to which itaconate is taken up from the extracellular environment in RA and what metabolic or inflammatory consequences this may have are yet to be determined. It is also unclear whether itaconate may have additional effects by acting at the cell surface. For example, the inhibition of succinate dehydrogenase has been attributed to the structural similarity of itaconate to succinate, allowing it to act as a competitive inhibitor of the succinate-consuming enzyme (132). This begs the question of whether extracellular itaconate can act as an antagonist of the succinate receptor SUCNR1 in a similar way.

## The NLRP3 Inflammasome

The term inflammasome describes a family of multi-protein complexes that have roles in cell survival and inflammation. These complexes are characterised by pattern recognition receptors that undergo oligomerisation and act as signalling hubs for the recruitment of the caspase-1 effector protein (142). One of the most well-studied of the inflammasomes, which we shall focus on here, is NLRP3. In addition to its roles in infection, cardiovascular disease, cancer and Alzheimer’s disease to name but a few, the NLRP3 inflammasome is widely studied in the context of inflammatory and autoimmune diseases (142–144). Gain-of-function mutations within NLRP3 are associated with the autoinflammatory disease cryopyrin-associated periodic syndrome (CAPS), and aberrant or excessive activation of NLRP3 has been linked to pathogenesis in SLE and various forms of arthritis (142, 144, 145). The NLRP3 inflammasome is also considered to be an important sensor of systemic metabolic disturbance, and is linked to the low-grade inflammation that plays a pathological role in metabolic disorders such as obesity and type 2 diabetes (146).

**Figure 3:**
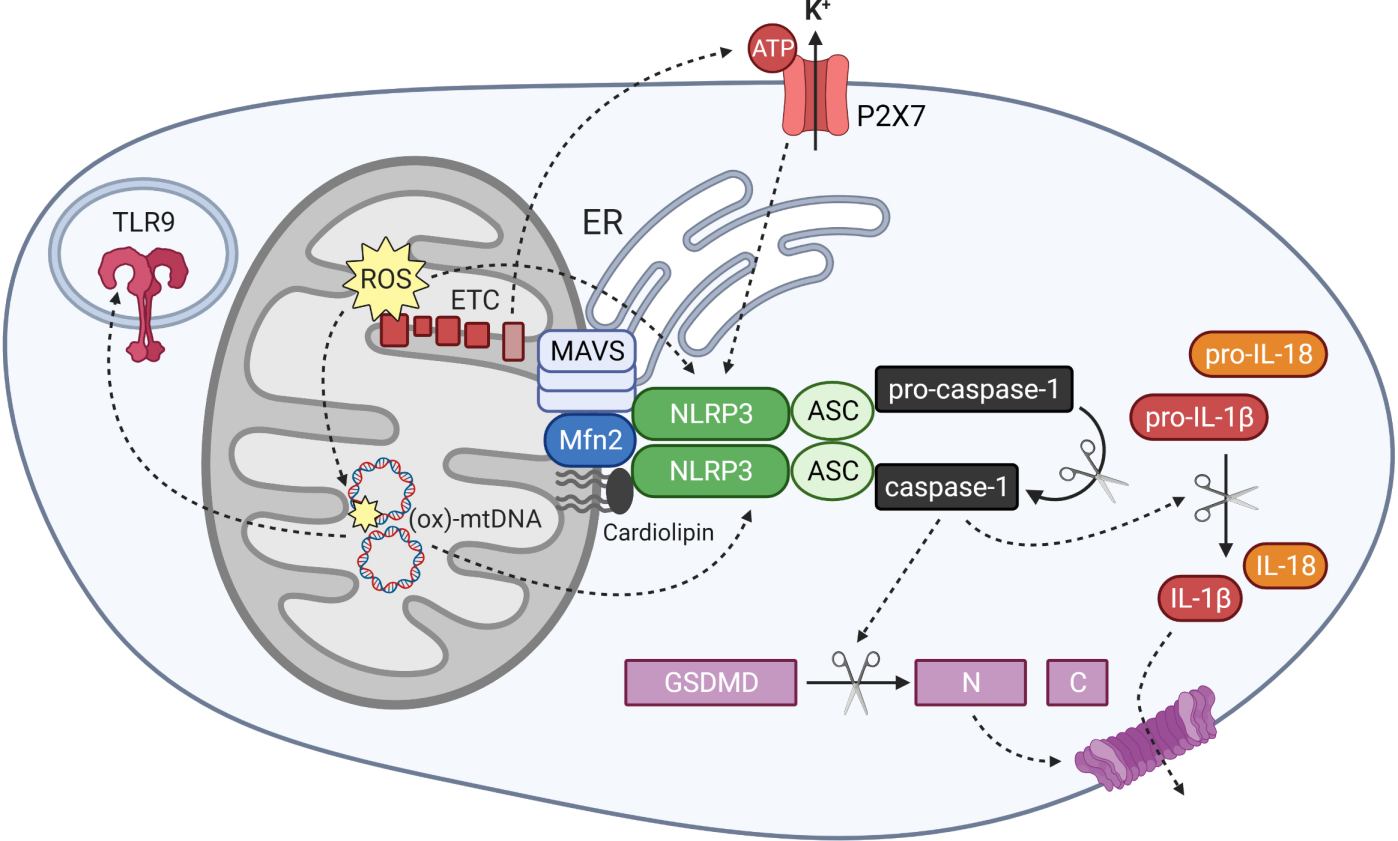
Mitochondrial regulation of NLRP3 inflammasome activation and mitochondrial DAMP activity. The NLRP3 inflammasome is activated by signals including mitochondrial reactive oxygen species (ROS), oxidised mitochondrial DNA (ox-mtDNA) and potassium efflux through the ATP-gated channel P2X7. Components of the inflammasome localise to mitochondrial and ER membranes upon activation, where they associate with MAVS, Mfn 2 and cardiolipin in the outer mitochondria membrane. Oligomerised NLRP3 and its adapter protein ASC recruit and activate caspase-1, which cleaves gasdermin D and interleukin-1 family cytokines into their active forms. Ox-mtDNA also exhibits DAMP activity by stimulating other pattern recognition receptors including the endosomal toll-like receptor TLR9.

NLRP3 is a nucleotide‐binding leucine‐rich repeat (NLR) receptor protein that is activated in response to a wide variety of stress signals, including bacterial, viral and fungal infections; endogenous damage-associated molecular patterns (DAMPs); cytokines; and lipid metabolites and other markers of metabolic stress (145, 146). Oligomerised NLRP3 recruits the adaptor protein ASC, and this in turn recruits procaspase-1, which undergoes self-cleavage to generate the active caspase-1 effector (**Figure 3**) (145). One outcome of NLRP3 inflammasome activation that underlies its link to inflammation, is the release of two members of the interleukin-1 family: IL-1β and IL-18. These cytokines are initially produced as extended precursor forms in response to an initial priming signal, for example stimulation of toll-like receptors and activation of classical inflammatory signalling pathways such as NF-κB. Subsequent release of the active cytokines requires their cleavage by activated caspase-1 (145). Another outcome of NLRP3 activation is a form of cell death termed pyroptosis, which also plays a role in propagating inflammation. Pyroptosis involves cleavage of the protein gasdermin D (GSDMD), which subsequently forms oligomeric pore structures within the plasma membrane (**Figure 3**). This allows release of inflammatory cytokines into the extracellular space, including IL-1β and IL-18, as well as cytoplasmic material that can further act as DAMPs and activate neighbouring immune cells (147).

Methods of activation and regulation of the NLRP3 inflammasome are extensively reviewed elsewhere (142, 145, 148). Here we shall focus solely on the mitochondria-linked mechanisms of regulation, and the relevance of NLRP3 activity to rheumatoid arthritis.

Mitochondria act as important stress-signalling organelles, and mitochondrial disruption results in NLRP3 activation *via* several mechanisms. Reactive oxygen species were identified as important mediators of NLRP3 activation, however the phagosome-located NADPH oxidase enzymes, which are major producers of ROS in myeloid cells, were shown not to be the source of the activating signal for NLRP3 (149–151). Dysfunctional mitochondria produce high levels of ROS, termed mtROS, through the transfer of electrons from the ETC to molecular oxygen (152). Artificial induction of high mtROS levels *in vitro* using ETC inhibitors resulted in production of active IL-1β, indicative of NLRP3 activation (151, 153, 154). Conversely, inhibition of ROS formation, or ROS scavenging by either endogenous or exogenous antioxidants, strongly impaired inflammasome activation and IL-1β release (151, 153, 154). Autophagic degradation and recycling of mitochondrial components by mitophagy is an important process for the removal of damaged or defective mitochondria. Inhibition of autophagy/mitophagy strongly elevated the cellular mtROS levels and was accompanied by an increase in IL-1β release, identifying mitophagy as an important process in preventing excessive inflammatory responses through intracellular mitochondrial stress (151, 154). Autophagy can also play another role in dampening the inflammatory response through direct destruction and recycling of inflammasome components, preventing cleavage and release of IL-1β and IL-18 (155).

In addition to ROS-dependent inflammasome activation, several groups have reported ROS-independent activation, and a requirement for functional mitochondria and an intact mitochondrial membrane potential has been described. It is likely that the precise activating signal dictates the method of inflammasome activation (19, 156, 157). Consistent with the role of the inflammasome as a metabolic sensor, fatty acid metabolism is linked to regulation of NLRP3 activation, and mitochondrial processes contribute to this regulation. The mitochondrial uncoupling protein UCP2 enhances NLRP3 and IL-1β expression by promoting fatty acid synthesis, which is achieved through increased expression of the enzyme fatty acid synthase. Increased fatty acid synthesis promotes activation of Akt and p38 MAPK signalling pathways, which drive NLRP3 and cytokine gene expression (158). Somewhat contradictory to these findings, fatty acid oxidation was also shown to promote NLRP3 activation, regulated by the mitochondrial fatty acid transport enzyme CPT1A and ROS generation by the enzyme NADPH oxidase 4, which also localises to mitochondria (159).

Un-activated NLRP3 protein resides in the cytoplasm, where it associates with the endoplasmic reticulum (ER). Upon priming and activation, the components of the inflammasome relocate to the mitochondria, where they co-localise with both mitochondria and ER at mitochondria-associated membrane (MAM) structures (151, 160). Several different mitochondrial components have been identified that interact with NLRP3 and facilitate the mitochondrial association and activation of the inflammasome. The mitochondrial-specific phospholipid cardiolipin associates directly with both NLRP3 and caspase-1, and was shown to be crucial for inflammasome activation in response to various stimuli (161, 162). NLRP3 also interacts with MAVS, an anti-viral protein that forms large aggregates within the outer mitochondrial membrane upon sensing of viral RNA. MAVS was found to recruit NLRP3 to the mitochondria and facilitate its activation in response to viral infection (160, 163). Mitofusin 2 (Mfn2), a protein involved in mitochondrial fusion, also interacts with NLRP3 and facilitates its activation in response to RNA viruses (19). Mfn2 is also known to associate with MAVS, suggesting that a large protein complex assembles at the mitochondrial surface and regulates inflammasome localisation and function (**Figure 3**) (19).

ATP is a well-recognised activator of NLRP3, and mitochondria represent an important source of ATP within the cell. Mitochondrial DNA (mtDNA) has also been implicated in inflammasome activation (164). These shall be discussed in more detail in a later section. Mitochondria therefore act as important signal generators, as well as signalling platforms, for the activation of the NLRP3 inflammasome.

Activity of the inflammasome has been shown to contribute to pathogenesis in RA. IL-1β has long been known to promote inflammatory and destructive processes within the RA synovium. This occurs through its actions on both immune cells and stromal cells, inducing the production of inflammatory cytokines, chemokines and adhesion molecules, as well as matrix-degrading enzymes and activators of osteoclast-mediated bone resorption (165). IL-1β also potently inhibits the tissue repair process, thereby exacerbating and prolonging joint damage (165). Infiltrating monocytes/macrophages are considered to be the major producers of IL-1β within the synovium, and NLRP3 was found to be activated within this cell population in RA synovial tissue samples (165, 166). Pharmacological inhibition of NLRP3 in the mouse CIA model significantly reduced disease severity and diminished both synovial inflammation and cartilage erosion (166). The ubiquitin-editing enzyme A20 (aka TNFAIP3) counteracts inflammatory signals and is important in the prevention of arthritis, as reviewed by Wu et al. (167). One mechanism by which A20 inhibits inflammation is through negative regulation of NLRP3 and caspase-1 activation, suppressing interleukin production and pyroptosis (167, 168). Myeloid-specific deletion of A20 results in a spontaneous polyarthritis with characteristics of RA, and deletion of NLRP3 could protect these mice from disease (168).

Due to the apparent importance of IL-1β action in RA, a recombinant IL-1 receptor antagonist, therapeutically named anakinra, was investigated in clinical trials. IL-1R antagonism showed significant clinical benefit in RA patients compared with placebo, and anakinra was approved for the treatment of conventional DMARD-resistant RA (169, 170). However, the results of these trails were less striking than was anticipated based on pre-clinical studies, and anakinra treatment showed efficacy in a lower proportion of patients in comparison to trials of other biologic DMARDs, such as anti-TNFα therapies (166, 170).

Interleukin-18, the second cytokine dependent upon inflammasome activation and cleavage by caspase-1, has also been implicated in driving pathogenic mechanisms in RA. Synovial tissue expression of IL-18 protein was found to correlate with CRP, and macrophages were also implicated in the production of this cytokine (171, 172). Deletion or therapeutic neutralisation of IL-18 reduced incidence and/or severity of disease in CIA mouse models, and the pathogenic roles of this cytokine include promoting polarisation and activation of Th1 cells and macrophages (171, 173, 174). Therefore, the direct targeting of the NLRP3 inflammasome may prove more effective in RA treatment than blocking IL-1 activity alone, due to inhibition of both IL-1β and IL-18 signalling, as well as preventing the release of alarmins *via* pyroptosis (166).

## Mitochondrial DAMPs

Mitochondria act as an important source of damage-associated molecular patterns (DAMPs). These endogenously derived molecules signal the occurrence of tissue injury, cellular destruction, or cellular stress, and can activate immune cells in a similar way to microbial-derived pathogen-associated molecular patterns (PAMPs). Due to the bacterial ancestry of mitochondria as described by the endosymbiont theory, mitochondrial components are able to activate the same pattern recognition receptors (PRRs) as exogenous PAMPs (175). DAMPs signal through several different receptors, including toll-like receptors (TLRs), NOD-like receptors (NLRs), RIG-I-like receptors (RLRs) and purinergic receptors (175). A large number of studies have focused on the role of the mitochondrion as a source of DAMPs and the potential involvement in the pathogenesis of RA.

### ATP

Mitochondria are the major cellular producers of ATP, with oxidative phosphorylation producing 18 times more ATP per glucose molecule than glycolysis. Release of ATP into the extracellular environment can occur as a result of cell death, and thus this small molecule acts as an alarmin, stimulating an immune response by acting on a variety of cell surface receptors (176). Extracellular ATP and UTP released from apoptotic cells act as chemoattractants for phagocytes such as monocytes, facilitating the removal of apoptotic cell debris. This is achieved through the activation of the purinergic G-protein coupled receptor P2Y2 on the cell surface of monocytes and macrophages (177). Early work in this field showed that *in vitro* treatment of human RA synovial fibroblasts with ATP or UTP was able to mobilise intracellular calcium, consistent with the activation of G-protein coupled receptors (GPCRs) such as P2Y2. Extracellular nucleotides and IL-1α synergised to stimulate synovial fibroblasts to secrete prostaglandins E_2_, an important lipid mediator of inflammatory signalling (178).

The most extensively studied link of extracellular ATP to inflammation is through its function as an activator of the NLRP3 inflammasome. ATP is commonly used *in vitro* as the second signal of inflammasome activation, as it promotes NLRP3 oligomerisation and cleavage of pro-IL-1β and pro-IL-18 (145). One mechanism by which ATP achieves activation of NLRP3 is through stimulation of the alternative purinergic receptor P2X7 (**Figure 3**). This is a ligand-gated ion channel that is broadly expressed, but which shows highest expression in the monocyte-macrophage lineage and has been extensively linked to regulation of the innate and adaptive immune systems (179, 180). Activation of the P2X7 receptor by ATP results in efflux of potassium ions, which is a common mechanism of NLRP3 activation that is also employed by the ionophore nigericin (145).

As discussed above, inflammasome activation and the upregulation of IL-1β and IL-18 production has been linked to RA disease processes, and the specific involvement of the P2X7 receptor has also been demonstrated. Elevated expression of the P2X7 receptor has been shown on total PBMCs and circulating monocytes from RA patients compared with control subjects, and RA patient blood cells produced significantly higher levels of IL-1β in response to LPS+ATP stimulation compared with healthy control cells (181–183). A recent study found that significantly elevated proportions of circulating Th17 and Th1 cells stained positive for P2X7 in samples from either SLE or RA patients compared with healthy controls. The percentage of P2X7-expressing Th17 cells correlated with both DAS28 and serum concentrations of IL-1β (184). Activation of the P2X7 receptor and NLRP3-dependent IL-1β production by dendritic cells is important for priming of T cells, and this pathway was shown to regulate CD8+ T cell activity in the anti-cancer immune response (185) and Th17 differentiation in arthritis models (181).

### Cytochrome c

Permeabilization of the outer mitochondrial membrane and release of cytochrome *c* from the intermembrane space into the cytosol is an important initiating signal for the intrinsic pathway of apoptosis. Cytosolic cytochrome *c* activates a caspase cleavage cascade that culminates in apoptotic cell death, which unlike other forms of cell death, is generally not inflammation-inducing (5, 186).

In contrast to its role in the cytosol, cytochrome *c* released into the extracellular environment may be able to act as a DAMP and stimulate immune cell activation (175, 186). Intra-articular injection of cytochrome *c* into mice resulted in a short-lasting inflammatory arthritis characterised by pronounced myeloid cell infiltration, in which neutrophils were the key drivers of pathology (187). Despite this link to arthritis symptoms, lower levels of cytochrome *c* were found in the serum of RA patients compared with healthy controls, and synovial levels were lower than matched serum samples. While the authors suggest that this may be due to increased consumption of cytochrome *c* in the inflammatory synovial environment, it remains to be seen whether there is a physiological role for the inflammation-promoting activity of this protein in RA (187). Extracellular cytochrome *c* may have relevance to autoimmunity in SLE, as autoantibodies to cytochrome *c* were detected in a small proportion of SLE patients in an early study (188).

### mtDNA

Mitochondrial DNA (mtDNA) consists of small circular DNA structures resembling plasmids that encode a limited number of genes, including ribosomal components, tRNAs, and key subunits of the ETC (189). These DNA molecules contain unmethylated CpG motifs similar to bacterial DNA, allowing them to act as agonists for PRRs including TLR9, cGAS and inflammasomes (189). MtDNA can be readily oxidised due to close proximity to the ETC machinery that acts as the site of mtROS production, and the oxidation state of mtDNA is an important factor in modulating its ability to activate PRRs (189, 190). The inflammation-promoting activity of mtDNA can be a cell intrinsic process through its release into the cytoplasm; or alternatively extracellular mtDNA can stimulate neighbouring cells or have wide-spread stimulatory effects through release into the circulation. The precise mechanisms of mtDNA DAMP activity, including proposed methods of release from the mitochondrion and receptor signalling pathways, are reviewed elsewhere (189, 191).

As a demonstration of the inflammatory properties of mtDNA, Collins et al. showed that intra-articular injection of mtDNA promoted inflammatory arthritis in mice, whereas injection of nuclear DNA had no effect. Pathology was driven by myeloid cells and could be ameliorated by inhibition of NF-κB activity (192). A synthetic oligodeoxynucleotide containing an oxidised residue was also able to induce inflammatory pathology, whereas the non-oxidised form of the same sequence had no inflammatory effect, demonstrating the greater immunostimulatory properties of oxidised DNA (192).

In the context of RA, extracellular mtDNA can be detected in synovial fluid and blood plasma (60, 190, 192). Extracellular mtDNA levels were found to be significantly higher in RA than in OA or healthy controls, and mtDNA copy number positively correlated with CRP (60). Levels of mtDNA bound to the surface of circulating blood cells were also elevated in RA, although there was no significant correlation with either disease activity or treatment response in this study (193). Hajizadeh et al. found that RA patients who tested positive for synovial fluid mtDNA were more likely also to be positive for rheumatoid factor, and that levels of 8-hydroxy-2’-deoxyguanosine (8-oxodG), a marker of oxidative DNA damage, were higher in RA synovial fluid than controls (190). These results suggest that oxidised mtDNA (ox-mtDNA), a potent PRR agonist, may contribute to immune cell activation in RA.

As discussed earlier, the synovial environment can be profoundly hypoxic, particularly in the presence of high levels of inflammation and dysregulated angiogenesis that can occur in RA and other forms of inflammatory arthritis (63, 64, 194). Low oxygen conditions cause increased production of mtROS, which can exacerbate oxidative damage of mtDNA and contribute to PRR activation (194). Biniecka et al. demonstrated that *in vitro* hypoxia exposure led to increased levels of 8-oxodG in a human synovial fibroblast cell line (63).

Neutrophils have been implicated as a source of extracellular mtDNA that contributes to inflammatory disease. Neutrophils from RA patients demonstrate an oxidative stress gene signature, and mtDNA levels have been shown to correlate with synovial neutrophil numbers (60). Direct stimulation of isolated neutrophils with mtDNA induced expression of RANKL, suggesting that this DAMP could drive osteoclast-mediated bone erosion in the RA joint (60). NETosis is a form of inducible cell death that occurs in neutrophils involving the release of complexes of decondensed chromatin and anti-microbial proteins, termed neutrophil extracellular traps (NETs). NETs function to trap and neutralise infectious agents, but are also implicated in the pathogenesis of autoimmune diseases including SLE and RA through stimulation of cytokine production and the release or extracellular generation of citrullinated proteins that act as autoantigens (57, 61, 195–197). Hypopolarised mitochondria have been shown to be released during NETosis, and ox-mtDNA contributes to NET formation in SLE, promoting interferon production and interferon stimulated gene signatures (61). In addition to promoting oxidation of mtDNA with immunostimulatory consequences, mitochondria-derived ROS are pivotal for the initiation of NETosis in response to certain triggers, as well as the spontaneous NETosis of pro-inflammatory low-density granulocytes from SLE patients (61, 198). Vorobjeva et al. also showed that the mitochondrial permeability transition pore is required for ROS production and NETosis in response to a calcium ionophore (198). NETosis-independent release of ox-mtDNA from neutrophils has also been demonstrated, which potently stimulates type I interferon production by plasmacytoid DCs, and this study found that some individuals with SLE develop antibodies against ox-mtDNA (59).

Cytosolic mtDNA has also been linked to the activation of the NLRP3 and AIM2 inflammasomes, resulting in caspase-1 activation and release of IL-1β and IL-18 (154, 164). The induction of apoptosis by inflammatory stimuli is associated with the release of mtDNA into the cytosol, and ox-mtDNA can interact with and activate NLRP3 in cells undergoing apoptosis (164). Inhibition of apoptosis, for example through the overexpression of the anti-apoptotic protein Bcl-2, could prevent NLRP3 activation and IL-1β release (164). Elevated levels of cytoplasmic ox-mtDNA have been detected in CD4+ T cells from RA patients. This was linked to reduced expression of the nuclease MRE11A, which forms part of the MRN DNA repair complex. Inhibition of this nuclease caused mitochondrial dysfunction, indicated by reduced oxidative metabolism and increased mtROS production. This led to enhanced oxidative damage of mtDNA and its release into the cytoplasm, activating the inflammasome and resulting in IL-1β release and pyroptotic cell death. Restoring MRE11A function in a chimeric mouse model of arthritis reduced ox-mtDNA levels, caspase-1 activation and synovial tissue inflammation (26). Reduced mtDNA repair capacity and mitochondrial damage has also been linked to chondrocyte apoptosis in osteoarthritic cartilage (199, 200).

The ability of other mitochondrial components to act as DAMPs has also been demonstrated. Cardiolipin, which is present in mitochondrial and bacterial membranes, has been shown to bind to TLR4 and either inhibit or activate signalling from this receptor, depending on the level of saturation or oxidation of the cardiolipin acyl chains (201). The relevance of these observations to autoimmune diseases has not yet been determined. Exposure of cardiolipin on the outer face of mitochondria also contributes to NLRP3 inflammasome activation, as previously discussed (161, 162, 201). Anti-cardiolipin antibodies can be detected in samples from RA patients, although these are more frequently associated with primary antiphospholipid syndrome or SLE (202), and this autoimmunity does not constitute true DAMP activity.

N-formyl peptides are also well-established mitochondria-derived DAMPs, which are a product of mitochondrial protein synthesis and act as strong chemoattractants for neutrophils (175). While an agonist of a formyl peptide receptor was shown to ameliorate disease in a serum transfer model of arthritis (203), this may be related to mimicking of endogenous anti- inflammatory agonists of this receptor rather than recapitulating mitochondrial N-formyl peptide detection, and the direct relevance of these mitochondrial-derived peptides to RA has not been investigated to our knowledge.

## mtDNA Mutation

In addition to its role as a DAMP, mitochondrial DNA is highly prone to mutation, which can promote inflammatory and autoimmune processes in a number of ways. The vulnerability of mtDNA to mutation is caused by the lack of extensive high fidelity repair processes, the high copy number of mtDNA within each cell, and the close proximity to ROS generating machinery (189, 204, 205). Mutations within the mitochondrial protein-coding genes can result in changes to peptide sequences that bring about mitochondrial dysfunction. Mutation of a mitochondrially-encoded subunit of the ETC may lead to increased ROS generation, which could subsequently exacerbate DNA mutation, as well as having other inflammatory consequences as discussed above (205). Mitochondrial peptides can activate the adaptive immune system through their presentation *via* MHC molecules. A change in the sequence of these peptides brought about by mtDNA mutation can result in breach of self-tolerance, leading to immune cell activation and the generation of autoantibodies (204–206). MtDNA mutation may also alter the ability of mtDNA to activate PRRs (189).

The frequency of mtDNA mutations was found to be significantly higher in the synovial tissue of patients with rheumatoid or psoriatic arthritis compared with OA and/or healthy individuals, and many of these mutations created amino acid changes (205, 207). MtDNA mutation frequency positively correlated with macroscopic synovitits and synovial levels of TNFα or IFNγ (207). *In vitro* exposure of immortalised RA fibroblasts to hypoxia (1% O_2_) increased the number mtDNA mutations, which could be prevented by treatment with antioxidants. This is consistent with hypoxia leading to increased oxidative stress and DNA oxidation. An increase in random mtDNA mutations showed negative correlation with synovial oxygen tension in patients with rheumatoid or psoriatic arthritis, suggesting that hypoxia-induced oxidative stress is likely a key driver of the elevated mutational burden in the hypoxic synovial environment (63). *In vitro* treatment of RA FLS with TNFα also led to increased mtDNA mutation frequency, indicating that in addition to mtDNA mutation promoting inflammation, an inflammatory environment also promotes mtDNA mutation (207). This damaging positive feedback loop may contribute to the persistent inflammatory response in RA.

As well as novel mutations arising as a result of inflammation or hypoxia, specific mitochondrial haplotypes have been linked to RA. The main variations found to be associated with the disease were within genes encoding components of the ETC (208). Rare single nucleotide variants of genomically-encoded ETC subunit or assembly factor genes were also found to associate with severe erosive RA (209).

## Targeting Mitochondria in RA Treatment

The therapeutic targeting of metabolic pathways initially emerged as a prospect for cancer treatment due to the relatively early discovery that metabolic reprogramming is essential to sustain cancer cell survival and proliferation. It is now apparent that targeting of metabolic processes, including mitochondrial modulation, could be therapeutically beneficial for a wide range of diseases including cardiovascular, neurodegenerative and autoimmune diseases (210, 211). As the importance of mitochondrial biology in inflammatory disease emerges, it is also becoming evident that many therapeutics currently in use for the treatment of RA have effects on mitochondrial activity, whether directly or indirectly. In some cases, these effects are known to contribute to the beneficial anti-inflammatory actions of the drugs, but can also be responsible for adverse effects of therapy. In other cases it remains to be seen whether the impact on mitochondrial function underlies the therapeutic efficacy.

### Methotrexate

Methotrexate (MTX) is a conventional synthetic disease-modifying anti-rheumatic drug (csDMARD) that is one of the first line therapeutics used for the treatment of RA and other forms of inflammatory arthritis, and which is also used in cancer treatment. MTX inhibits the mitochondrial folate pathway by acting as a competitive inhibitor of folate-dependent enzymes such as dihydrofolate reductase (DHFR), due to the structural similarity of MTX to folic acid. This results in reduced synthesis of purines and pyrimidines, thus bringing about anti-proliferative effects (212). Whilst this was the main intended target pathway of MTX, it is now thought that additional mechanisms are responsible for many of the anti-inflammatory effects of this drug. This is reviewed by Bedoui et al. (212).

Elevated ROS production is an important mechanism by which MTX affects cellular function and survival, and mitochondria have been shown to contribute to this ROS generation (213, 214). Elevated ROS can propagate cellular oxidative stress, which contributes to the anti-inflammatory or anti-cancer actions of the drug, for example through induction of T cell apoptosis (213, 215). Lee et al. found that responsiveness to MTX therapy in RA patients was linked to susceptibility of the patient’s FLS to mitochondrial depolarisation and apoptosis in response to the drug (216).

However, oxidative stress and mitochondrial dysfunction have also been linked to common adverse effects of MTX therapy. Various combinations of lipid peroxidation, mitochondrial depolarisation, respiratory chain inhibition, reduced ATP levels, cytochrome *c* release and mitochondrial swelling have been reported in liver, kidney, small intestine and platelets following either *in vivo* or *in vitro* MTX treatment (214, 217–219). Hepatoxicity is one of the most common adverse effects of MTX therapy, however severe liver injury is more commonly associated with high dose therapy used in cancer treatment rather than the low doses used in the treatment of inflammatory diseases (212). Thrombocytopenia can be experienced by cancer or RA patients after long-term MTX therapy, which may be due to oxidative stress and apoptosis of platelets (214).

In contrast, *in vitro* MTX treatment was found to increase respiratory rate and respiratory capacity in the contexts of B cell lymphoma and breast cancer. This was due to AMPK activation through the accumulation of the purine synthesis intermediate AICAR, which was shown to contribute to the therapeutic effects of MTX in cancer (220). AMPK activation also has therapeutic potential for inflammatory diseases, which shall be discussed below.

### Leflunomide

Leflunomide is also an approved csDMARD that is indicated for the treatment of adults with RA, although its use is not as common as other csDMARDs due to the risk of hepatic toxicity (221). Mitochondrial disruption has been implicated in this hepatotoxicity, as the drug caused ATP depletion and mitochondrial depolarisation in a hepatocellular carcinoma cell line. These effects were linked to ER stress and inhibition of ATP synthase activity, resulting in cytotoxicity (222).

Leflunomide inhibits the mitochondrial inner membrane protein dihydroorotate dehydrogenase (DHODH), reducing the *de novo* synthesis of pyrimidines and thus inhibiting proliferation. Pyrimidine depletion by leflunomide was recently shown to promote enhanced expression of mitofusins 1 and 2 (Mfn1/2), resulting in increased mitochondrial fusion (223, 224). Leflunomide and its active metabolite teriflunomide also inhibit the activity of ETC complex III, which may be explained by coupling of the pyrimidine synthesis pathway to ETC activity through DHODH-mediated reduction of ubiquinol (223, 225). DHODH inhibition in T cells preferentially reduced proliferation of high affinity antigen-specific T cells, due to their high demand for oxidative metabolism in the early stages of proliferation. In this way the drug is thought to inhibit autoreactive T cell expansion in autoimmune diseases such as multiple sclerosis and RA (225). As complex III inhibition is known to result in mtROS production, and DHODH silencing has been shown to increase ROS (226), it is possible that leflunomide brings about apoptosis induction by oxidative stress, similarly to MTX.

### Sulfasalazine

Sulfasalazine is another csDMARD that has been used by rheumatologists for decades, but a full understanding of its anti-inflammatory mechanisms of action is lacking. It has been reported that sulfasalazine inhibits *de novo* purine synthesis, resulting in adenosine release that contributes to the anti-inflammatory activity of this drug, similarly to MTX (227). Sulfasalazine has anti-proliferative and cytotoxic effects, making it an effective therapeutic for cancer and inflammatory disease treatment, and it induces apoptosis of T cells through mitochondrial permeabilization (228). Ferroptosis is a form of cell death characterised by increased mitochondrial membrane densities and outer membrane rupture, which is associated with excessive ROS production through iron metabolism (229). These characteristics were demonstrated in response to sulfasalazine treatment in lymphoma and breast cancer cells *in vitro*, and inhibition of plasma membrane cystine/glutamate antiporters was implicated in sulfasalazine-induced ferroptosis (229–231).

Mitochondrial disruption has also been implicated in renal injury caused by sulfasalazine. High doses of the drug caused oxidative stress in rat kidneys, demonstrated by increased ROS, lipid peroxidation and decreased levels of reduced glutathione, which were associated with mitochondrial depolarisation and swelling (232).

### Biologics

The development of targeted biologic therapies for rheumatoid diseases has revolutionised RA treatment and significantly improved quality of life for many people living with this disease. Whilst none of these therapeutics are designed to target the mitochondrion directly, many biologics block cytokine signalling, and it has been widely demonstrated that inflammatory cytokine signalling has robust effects on mitochondrial biology. For example, treatment with TNFα blocking therapy resulted in reduced frequency of mitochondrial mutations and decreased markers of oxidative stress in the synovia of patients who clinically responded to the therapy (233). Several other studies have shown reductions in reactive oxygen and nitrogen species and increases in antioxidants in response to anti-TNFα therapy (234). Reduced oxidative stress has also been demonstrated in patients treated with tocilizumab, an antibody directed against the IL-6 receptor, suggesting that combating cytokine-induced ROS production may be an important therapeutic mechanism (234).

Gene expression analyses have been used to interrogate the effects of RA therapies on immune cells. Meugnier et al. profiled gene expression changes in PBMCs from RA patients following clinical anti-TNFα treatment and found enrichment of genes related to oxido-reduction and electron transfer. This included upregulation of several ETC subunit genes and mitochondrial ribosomal components (235). Derambure et al. analysed whole blood RNA of patients treated with abatacept. This biologic is a fusion of the extracellular domain of CTLA-4 and the Fc portion of IgG1, which disrupts T cell co-stimulation through CD28 and affects antigen presenting cell signalling. Pre-therapy expression of several ETC subunit genes was significantly lower in patients who responded to abatacept plus MTX therapy compared with non-responders, and five of these genes were significantly upregulated following six months of therapy in the responder group (236). These findings suggest that an increase in respiratory chain activity may play a role in the therapeutic action of diverse biologic drugs.

### JAK/STAT Inhibitors

The JAK/STAT inhibitors are a class of small molecule targeted synthetic DMARDs that are relatively new to the arsenal of therapeutics used by rheumatologists, offering a useful alternative to biologic therapies. The JAK family of tyrosine kinases are activated downstream of several different cytokine receptors and signal through activation of the STAT family of transcription factors [reviewed in (237)]. Tofacitinib, a pan-JAK inhibitor that is approved for the treatment of rheumatoid and psoriatic arthritis, increased expression of several mitochondrial genes and led to increased oxidative respiration rate and ATP synthesis in RA FLS, while also decreasing ROS levels and glycolytic enzyme expression (238). STAT3 activation has previously been linked to hypoxia and promotes HIF-1α activity, which may be an important mechanism by which JAK/STAT inhibition can regulate metabolic processes during inflammation (66). Interestingly, tofacitinib also increased lipolysis, mitochondrial activity and uncoupling protein 1 (UCP1) expression in adipocytes, which was suggested to be of therapeutic interest in obesity and may help to combat the systemic metabolic complications of inflammatory diseases (239).

### Future Therapeutic Opportunities

Despite the array of therapeutics that are already approved for the treatment of RA, some patients fail to respond to any of these treatments, and sustained clinical remission is achieved in fewer than half of patients (240, 241). These facts highlight the continued need for additional therapies that are tailored to an individual patient’s disease. Due to the mounting evidence of metabolic involvement in disease, metabolic targets are coming to the forefront in the search for novel therapeutic strategies. The wide array of potential metabolic targets for treatment of autoimmune disease are reviewed by Piranavan et al. (211).

A pathway that has gained much attention in this area is the energy and metabolite-sensing system, coordinated by the opposing actions of AMPK and mTOR. This system plays a number of roles in the regulation of immune cell function, including cell fate and survival decisions in lymphocytes (242). Complex crosstalk exists between these two sensors and the mitochondria, with each influencing the activity of the others. Inhibition of mTOR activity with compounds such as rapamycin or everolimus, which are used to prevent transplant rejection and in cancer therapy, has shown therapeutic benefit in clinical trials to treat multiple different autoimmune conditions (243).

An alternative approach is the activation of AMPK, which itself inhibits mTOR. This can be achieved using metformin, a drug used in the management of type 2 diabetes, which inhibits complex I of the ETC, thereby reducing mitochondrial activity and ATP generation. The resultant increase in AMP/ATP ratio causes AMPK activation, bringing about inhibition of anabolic metabolism and cellular proliferation. AMPK activation also downregulates several inflammatory signalling pathways such as NF-κB and JAK-STAT pathways, and as a result has broad anti-inflammatory actions (244). Efficacy of metformin has been shown in several pre-clinical models of arthritis, associated with decreased inflammatory cytokine production, reduced Th17 numbers and increased Tregs, and inhibition of osteoclastogenesis, amongst other effects (244–247). The examination of metformin use specifically for RA in clinical trials is currently lacking, but this drug is generally well-tolerated and could have the added benefit of reducing the systemic metabolic complications that can be associated with RA, such as obesity and diabetes, due to its effects on glucose homeostasis (244). However, Wen et al. reported that *ex vivo* treatment of RA patient T cells with metformin was unable to oppose inflammatory T cell differentiation due to impairment of the AMPK activation pathway in these cells. This suggests that direct targeting of mTOR activity may be a better therapeutic option than AMPK activation for human RA disease (27).

Another possible therapeutic strategy is the targeting of ROS production, as ROS and oxidative stress contribute to inflammatory processes and RA pathogenesis in multiple ways, as discussed throughout this review. Metformin inhibits oxidative stress through upregulation of antioxidant enzymes and downregulation of the ROS producer NADPH oxidase, and these effects are thought to contribute to the anti-inflammatory and anti-fibrotic effects of this drug (244, 248, 249). A mitochondrially-targeted antioxidant showed modest therapeutic benefit in a rat CIA model, suggesting that mitochondrial ROS production could be a viable target (250). The targeting of ROS systems must be approached with caution, however. In contrast to pathological roles of ROS, reduced ROS signalling downstream of TCR activation is implicated in hyperproliferation and inflammatory differentiation of RA T cells, as discussed earlier (44). The induction of ROS generation and ROS-induced apoptosis appears to be a mechanism by which current csDMARDs such as MTX mediate their therapeutic effects, although this also contributes to adverse effects through apoptosis of other cell types. It has been suggested that antioxidant treatment could be used in combination with other therapeutics, either to enhance efficacy or reduce side effects, but this approach requires further validation and will depend on the specific mechanism of action of the drug in question (251, 252).

As discussed above, the NLRP3 inflammasome, which is regulated by mitochondria and acts as an important sensor and signal disseminator for both metabolic and infectious disturbances, is also a potentially viable future target that is being investigated for multiple conditions (148, 166). Preventing NLRP3 activation by inhibiting activation of ATP-gated ion channels such as P2X7 has been explored for its potential in RA treatment (179, 253). P2X7-KO mice showed significantly reduced incidence and severity of collagen antibody-induced arthritis (180). *In vivo* treatment with P2X7 antagonists suppressed inflammation and reduced disease severity in both a mouse collagen antigen-induced arthritis model (181) and a rat streptococcal cell wall arthritis model (253). However, in two clinical trials of small molecule P2X7 antagonists, targeting of this receptor showed no clinical benefit over placebo in RA patients who had shown no response to first line conventional DMARDs (254, 255). This therapy therefore showed less efficacy than directly targeting IL-1β itself with anakinra (170), suggesting that mechanisms in addition to ATP signalling through this receptor contribute to NLRP3 activation and IL-1β production in RA disease. It remains to be seen whether targeting of extracellular ATP signalling can prove beneficial in other autoimmune conditions (179).

Overall, there is plenty of scope for the targeting of metabolic and mitochondrial pathways for the treatment of autoimmune and inflammatory diseases such as RA, particularly as more is learnt about the contribution of these processes to the immune response and disease pathogenesis.

## Conclusions and Outstanding Questions

We have made great strides in recent years in our understanding of the contributions made by metabolic regulation to immune cell function and disease processes in autoimmunity. Through their central role in metabolism and their ability to act as signalling hubs, mitochondria influence numerous cellular functions and can module immune cell activity. The capacity of mitochondrial components to directly activate immune receptors can also have potentially damaging consequences, however whether these signals act as initiating stimuli in RA disease is yet to be fully established.

Despite the progress already made, there remain many unanswered questions regarding the precise roles of mitochondria in driving disease in RA. Several of these have been highlighted in the body of this review, including the impacts of hypoxia and nitric oxide on mitochondrial ATP production in the synovium, the precise signalling roles of mitochondria-derived metabolites, and the differences in metabolic commitments between different subpopulations of synovial cells.

One of the major gaps in our understanding of metabolic regulation is the lack of data from the site of disease manifestation – the synovial joint itself. This has been due to the technical complexities involved in accessing and studying the very small numbers of cells from this environment, but this analysis will be crucial in determining whether direct targeting of metabolic and mitochondrial pathways will have real therapeutic benefit. Advances in single cell and *in situ* imaging technologies will be very helpful in answering these questions and will add to existing data from circulating immune cells. In addition, many studies that have looked at fibroblast-like synoviocyte biology have done so using tissue from joint replacements, which represents very late-stage disease. These cells may not bear much resemblance to FLS at the initiation of disease, with the latter likely to give more useful information about disease causes and potential opportunities for early intervention to prevent disease progression.

Another gap in our knowledge is the understanding of mitochondrial function in other cell types that contribute to RA disease, such as B cells and neutrophils, as the majority of the work in this field has focused on T cells and macrophages. This mirrors the landscape of the immunometabolism field in general, however the availability of large single cell gene expression datasets that cover multiple synovial cell populations should aid in the more rapid expansion of these areas in a disease-relevant manner (256). The metabolic communication between these cell populations is also an intriguing and important consideration, as no cell exists in isolation. This communication may constitute competition for, or exchange of, metabolites in the synovial environment. A more extreme form of metabolic communication is the exchange of whole mitochondria between cells, which has been demonstrated in other contexts including tumour models, lung inflammation and wound healing (257, 258). Whether this occurs in RA is currently unknown.

The precise regulation of mitochondrial architecture, including fusion/fission, supercomplex organisation and respiratory chain composition, also requires further investigation in the context of inflammatory disease. These aspects help to control ATP production, oxidative stress and optimisation of substrate usage, factors that can all impact upon immune cell function. Mitochondrial architecture is known to be regulated by hypoxia and inflammatory signals in other contexts (259–261).

The impact of therapy on mitochondrial function is apparent for several currently used therapeutics, and it will be interesting to see whether other anti-rheumatic drugs in use or development similarly affect this organelle. The targeting of metabolic processes has become an attractive prospect for novel therapeutic development, as well as for the re-purposing of treatments from other diseases for use in inflammatory and autoimmune conditions, for example mTOR and AMPK modulators. However, a more complete understanding of the nuances of mitochondrial function in different cell types in RA is required before the opportunities for targeting mitochondrial pathways can be fully realised. Since metabolic and mitochondrial activities are essential for all cell and tissue types, an important consideration will be to achieve therapeutic benefit without unacceptable adverse effects.

## Author Contributions

SC and AC devised, and SC wrote the manuscript. LM contributed to figure preparation. MK-S and AC reviewed and edited the manuscript. All authors contributed to the article and approved the submitted version.

## Funding

The authors’ work is supported by the Research into Inflammatory Arthritis Centre Versus Arthritis (grant 22072) and by Versus Arthritis grants 21802 (AC and MK-S) and 22272 (MK-S).

## Conflict of Interest

The authors declare that the research was conducted in the absence of any commercial or financial relationships that could be construed as a potential conflict of interest.

## References

[1] Firestein GS McInnes IB. Immunopathogenesis of Rheumatoid Arthritis. Immunity (2017) 46(2):183–96. 10.1016/j.immuni.2017.02.006 PMC5385708 28228278

[2] Wu CY Yang HY Luo SF Lai JH. From Rheumatoid Factor to Anti-Citrullinated Protein Antibodies and Anti-Carbamylated Protein Antibodies for Diagnosis and Prognosis Prediction in Patients With Rheumatoid Arthritis. Int J Mol Sci (2021) 22(2):18. 10.3390/ijms22020686 PMC7828258 33445768

[3] Lewis MJ Barnes MR Blighe K Goldmann K Rana S Hackney JA. Molecular Portraits of Early Rheumatoid Arthritis Identify Clinical and Treatment Response Phenotypes. Cell Rep (2019) 28(9):2455–+. 10.1016/j.celrep.2019.07.091 PMC6718830 31461658

[4] Orange DE Agius P DiCarlo EF Robine N Geiger H Szymonifka J. Identification of Three Rheumatoid Arthritis Disease Subtypes by Machine Learning Integration of Synovial Histologic Features and RNA Sequencing Data. Arthritis Rheumatol (2018) 70(5):690–701. 10.1002/art.40428 29468833 PMC6336443

[5] Pena-Blanco A Garcia-Saez AJ. Bax, Bak and Beyond - Mitochondrial Performance in Apoptosis. FEBS J (2018) 285(3):416–31. 10.1111/febs.14186 28755482

[6] Baier A Meineckel I Gay S Pap T. Apoptosis in Rheumatoid Arthritis. Curr Opin Rheumatol (2003) 15(3):274–9. 10.1097/00002281-200305000-00015 12707581

[7] Malemud CJ. Defective T-Cell Apoptosis and T-Regulatory Cell Dysfunction in Rheumatoid Arthritis. Cells (2018) 7(12):10. 10.3390/cells7120223 PMC6316166 30469466

[8] Rambold AS Pearce EL. Mitochondrial Dynamics At the Interface of Immune Cell Metabolism and Function. Trends Immunol (2018) 39(1):6–18. 10.1016/j.it.2017.08.006 28923365

[9] Buck MD O’Sullivan D Geltink RIK Curtis JD Chang CH Sanin DE. Mitochondrial Dynamics Controls T Cell Fate Through Metabolic Programming. Cell (2016) 166(1):63–76. 10.1016/j.cell.2016.05.035 27293185 PMC4974356

[10] Kapetanovic R Afroz SF Ramnath D Lawrence G Okada T Curson JEB. Lipopolysaccharide Promotes Drp1-dependent Mitochondrial Fission and Associated Inflammatory Responses in Macrophages. Immunol Cell Biol (2020) 98(7):528–39. 10.1111/imcb.12363 PMC7497224 32686869

[11] Lee J Choi JA Cho SN Son SH Song CH. Mitofusin 2-Deficiency Suppresses Mycobacterium Tuberculosis Survival in Macrophages. Cells (2019) 8(11):14. 10.3390/cells8111355 PMC6912353 31671648

[12] Duroux-Richard I Roubert C Ammari M Presumey J Grun JR Haupl T. miR-125b Controls Monocyte Adaptation to Inflammation Through Mitochondrial Metabolism and Dynamics. Blood (2016) 128(26):3125–36. 10.1182/blood-201602-697003 PMC5335801 27702798

[13] Lachmandas E Boutens L Ratter JM Hijmans A Hooiveld GJ Joosten LAB. Microbial Stimulation of Different Toll-like Receptor Signalling Pathways Induces Diverse Metabolic Programmes in Human Monocytes. Nat Microbiol (2017) 2(3):10. 10.1038/nmicrobiol.2016.246 27991883

[14] Wu B Goronzy JJ Weyand CM. Metabolic Fitness of T Cells in Autoimmune Disease. Immunometabolism (2020) 2(2):e200017. 10.20900/immunometab20200017 32477606 PMC7261019

[15] Dakin SG Coles M Sherlock JP Powrie F Carr AJ Buckley CD. Pathogenic Stromal Cells as Therapeutic Targets in Joint Inflammation. Nat Rev Rheumatol (2018) 14(12):714–26. 10.1038/s41584-018-0112-7 30420750

[16] Kim EK Kwon JE Lee SY Lee EJ Kim DS Moon SJ. Il-17-mediated Mitochondrial Dysfunction Impairs Apoptosis in Rheumatoid Arthritis Synovial Fibroblasts Through Activation of Autophagy. Cell Death Dis (2017) 8:10. 10.1038/cddis.2016.490 PMC5386390 28102843

[17] Wang XY Chen ZF Fan XM Li W Qu JQ Dong C. Inhibition of DNM1L and Mitochondrial Fission Attenuates Inflammatory Response in Fibroblast-Like Synoviocytes of Rheumatoid Arthritis. J Cell Mol Med (2020) 24(2):1516–28. 10.1111/jcmm.14837 PMC6991664 31755231

[18] Patten DA Wong J Khacho M Soubannier V Mailloux RJ Pilon-Larose K. OPA1-Dependent Cristae Modulation is Essential for Cellular Adaptation to Metabolic Demand. EMBO J (2014) 33(22):2676–91. 10.15252/embj.201488349 PMC4282575 25298396

[19] Ichinohe T Yamazaki T Koshiba T Yanagi Y. Mitochondrial Protein Mitofusin 2 is Required for NLRP3 Inflammasome Activation After RNA Virus Infection. Proc Natl Acad Sci USA (2013) 110(44):17963–8. 10.1073/pnas.1312571110 PMC3816452 24127597

[20] Bordt EA Clerc P Roelofs BA Saladino AJ Tretter L Adam-Vizi V. The Putative Drp1 Inhibitor Mdivi-1 Is a Reversible Mitochondrial Complex I Inhibitor That Modulates Reactive Oxygen Species. Dev Cell (2017) 40(6):583–+. 10.1016/j.devcel.2017.02.020 PMC5398851 28350990

[21] Ploumi C Daskalaki I Tavernarakis N. Mitochondrial Biogenesis and Clearance: A Balancing Act. FEBS J (2017) 284(2):183–95. 10.1111/febs.13820 27462821

[22] Ichimiya T Yamakawa T Hirano T Yokoyama Y Hayashi Y Hirayama D. Autophagy and Autophagy-Related Diseases: A Review. Int J Mol Sci (2020) 21(23):21. 10.3390/ijms21238974 PMC7729615 33255983

[23] Chadha S Behl T Bungau S Kumar A Kaur R Venkatachalam T. Focus on the Multimodal Role of Autophagy in Rheumatoid Arthritis. Inflammation (2021) 12:1-12. 10.1007/s10753-020-01324-8 32954452

[24] Yang Z Fujii H Mohan SV Goronzy JJ Weyand CM. Phosphofructokinase Deficiency Impairs ATP Generation, Autophagy, and Redox Balance in Rheumatoid Arthritis T Cells. J Exp Med (2013) 210(10):2119–34. 10.1084/jem.20130252 PMC3782046 24043759

[25] Ansari MY Khan NM Ahmad I Haqqi TM. Parkin Clearance of Dysfunctional Mitochondria Regulates ROS Levels and Increases Survival of Human Chondrocytes. Osteoarthr Cartil (2018) 26(8):1087–97. 10.1016/j.joca.2017.07.020 PMC5803469 28801211

[26] Li YY Shen Y Jin K Wen ZK Cao WQ Wu BW. The DNA Repair Nuclease Mre11a Functions as a Mitochondrial Protector and Prevents T Cell Pyroptosis and Tissue Inflammation. Cell Metab (2019) 30(3):477–+. 10.1016/j.cmet.2019.06.016 PMC7093039 31327667

[27] Wen Z Jin K Shen Y Yang Z Li Y Wu B. N-Myristoyltransferase Deficiency Impairs Activation of Kinase AMPK and Promotes Synovial Tissue Inflammation. Nat Immunol (2019) 20(3):313–25. 10.1038/s41590-018-0296-7 PMC6396296 30718913

[28] Herzig S Shaw RJ. AMPK: Guardian of Metabolism and Mitochondrial Homeostasis. Nat Rev Mol Cell Biol (2018) 19(2):121–35. 10.1038/nrm.2017.95 PMC5780224 28974774

[29] Morita M Gravel SP Hulea L Larsson O Pollak M St-Pierre J. mTOR Coordinates Protein Synthesis, Mitochondrial Activity and Proliferation. Cell Cycle (2015) 14(4):473–80. 10.4161/15384101.2014.991572 PMC4615141 25590164

[30] Chandel NS Jeffs P. Navigating Metabolism. Cold Spring Harbor Laboratory Press (2015).

[31] Falconer J Murphy AN Young SP Clark AR Tiziani S Guma M. Synovial Cell Metabolism and Chronic Inflammation in Rheumatoid Arthritis. Arthritis Rheumatol (2018) 70(7):984–99. 10.1002/art.40504 PMC6019623 29579371

[32] O’Neill LAJ Kishton RJ Rathmell J. A Guide to Immunometabolism for Immunologists. Nat Rev Immunol (2016) 16(9):553–65. 10.1038/nri.2016.70 PMC5001910 27396447

[33] Van den Bossche J O’Neill LA Menon D. Macrophage Immunometabolism: Where Are We (Going)? Trends Immunol (2017) 38(6):395–406. 10.1016/j.it.2017.03.001 28396078

[34] Diskin C Palsson-McDermott EM. Metabolic Modulation in Macrophage Effector Function. Front Immunol (2018) 9:270. 10.3389/fimmu.2018.00270 29520272 PMC5827535

[35] Ryan DG O’Neill LAJ. Krebs Cycle Rewired for Macrophage and Dendritic Cell Effector Functions. FEBS Lett (2017) 591(19):2992–3006. 10.1002/1873-3468.12744 28685841

[36] Vijayan V Pradhan P Braud L Fuchs HR Gueler F Motterlini R. Human and Murine Macrophages Exhibit Differential Metabolic Responses to Lipopolysaccharide - A Divergent Role for Glycolysis. Redox Biol (2019) 22:9. 10.1016/j.redox.2019.101147 PMC6396203 30825774

[37] Zeisbrich M Yanes RE Zhang H Watanabe R Li YY Brosig L. Hypermetabolic Macrophages in Rheumatoid Arthritis and Coronary Artery Disease Due to Glycogen Synthase Kinase 3b Inactivation. Ann Rheum Dis (2018) 77(7):1053–62. 10.1136/annrheumdis-2017-212647 PMC6589337 29431119

[38] Yamashita T Hagino H Hayashi I Hayashibara M Tanida A Nagira K. Effect of a Cathepsin K Inhibitor on Arthritis and Bone Mineral Density in Ovariectomized Rats With Collagen-Induced Arthritis. Bone Rep (2018) 9:1–10. 10.1016/j.bonr.2018.05.006 29992179 PMC6034140

[39] Alivernini S MacDonald L Elmesmari A Finlay S Tolusso B Gigante MR. Distinct Synovial Tissue Macrophage Subsets Regulate Inflammation and Remission in Rheumatoid Arthritis. Nat Med (2020) 26(8):1295–306. 10.1038/s41591-020-0939-8 32601335

[40] Culemann S Gruneboom A Nicolas-Avila JA Weidner D Lammle KF Rothe T. Locally Renewing Resident Synovial Macrophages Provide a Protective Barrier for the Joint. Nature (2019) 572(7771):670–+. 10.1038/s41586-019-1471-1 PMC6805223 31391580

[41] Huang QQ Doyle R Chen SY Sheng Q Misharin AV Mao Q. Critical Role of Synovial Tissue-Resident Macrophage Niche in Joint Homeostasis and Suppression of Chronic Inflammation. Sci Adv (2021) 7(2):eabd0515. 10.1126/sciadv.abd0515 33523968 PMC7787490

[42] O’Neill LAJ Pearce EJ. Immunometabolism Governs Dendritic Cell and Macrophage Function. J Exp Med (2016) 213(1):15–23. 10.1084/jem.20151570 26694970 PMC4710204

[43] Pucino V Certo M Bulusu V Cucchi D Goldmann K Pontarini E. Lactate Buildup At the Site of Chronic Inflammation Promotes Disease by Inducing Cd4(+) T Cell Metabolic Rewiring. Cell Metab (2019) 30(6):1055–+. 10.1016/j.cmet.2019.10.004 PMC6899510 31708446

[44] Yang Z Shen Y Oishi H Matteson EL Tian L Goronzy JJ. Restoring Oxidant Signaling Suppresses Proarthritogenic T Cell Effector Functions in Rheumatoid Arthritis. Sci Transl Med (2016) 8(331):331ra38. 10.1126/scitranslmed.aad7151 PMC5074090 27009267

[45] Wu BW Qiu JT Zhao TTV Wang YN Maeda T Goronzy IN. Succinyl-Coa Ligase Deficiency in Pro-inflammatory and Tissue-Invasive T Cells. Cell Metab (2020) 32(6):20. 10.1016/j.cmet.2020.10.025 PMC7755381 33264602

[46] Souto-Carneiro MM Klika KD Abreu MT Meyer AP Saffrich R Sandhoff R. Effect of Increased Lactate Dehydrogenase A Activity and Aerobic Glycolysis on the Proinflammatory Profile of Autoimmune Cd8+ T Cells in Rheumatoid Arthritis. Arthritis Rheumatol (2020) 72(12):2050–64. 10.1002/art.41420 32602217

[47] Garcia-Carbonell R Divakaruni AS Lodi A Vicente-Suarez I Saha A Cheroutre H. Critical Role of Glucose Metabolism in Rheumatoid Arthritis Fibroblast-like Synoviocytes. Arthritis Rheumatol (2016) 68(7):1614–26. 10.1002/art.39608 PMC4963240 26815411

[48] Matsui T Nakata N Nagai S Nakatani A Takahashi M Momose T. Inflammatory Cytokines and Hypoxia Contribute to F-18-FDG Uptake by Cells Involved in Pannus Formation in Rheumatoid Arthritis. J Nucl Med (2009) 50(6):920–6. 10.2967/jnumed.108.060103 19443596

[49] Bustamante MF Oliveira PG Garcia-Carbonell R Croft AP Smith JM Serrano RL. Hexokinase 2 as a Novel Selective Metabolic Target for Rheumatoid Arthritis. Ann Rheum Dis (2018) 77(11):1636–43. 10.1136/annrheumdis-2018-213103 PMC6328432 30061164

[50] Shin YJ Han SH Kim DS Lee GH Yoo WH Kang YM. Autophagy Induction and CHOP Under-Expression Promotes Survival of Fibroblasts From Rheumatoid Arthritis Patients Under Endoplasmic Reticulum Stress. Arthritis Res Ther (2010) 12(1):R19. 10.1186/ar2921 20122151 PMC2875648

[51] Xu K Xu P Yao JF Zhang YG Hou WK Lu SM. Reduced Apoptosis Correlates With Enhanced Autophagy in Synovial Tissues of Rheumatoid Arthritis. Inflamm Res (2013) 62(2):229–37. 10.1007/s00011-012-0572-1 23178792

[52] Croft AP Campos J Jansen K Turner JD Marshall J Attar M. Distinct Fibroblast Subsets Drive Inflammation and Damage in Arthritis. Nature (2019) 570(7760):246–+. 10.1038/s41586-019-1263-7 PMC6690841 31142839

[53] Rao DA Gurish MF Marshall JL Slowikowski K Fonseka CY Liu YY. Pathologically Expanded Peripheral T Helper Cell Subset Drives B Cells in Rheumatoid Arthritis. Nature (2017) 542(7639):110–+. 10.1038/nature20810 PMC5349321 28150777

[54] Caro-Maldonado A Wang R Nichols AG Kuraoka M Milasta S Sun LD. Metabolic Reprogramming is Required for Antibody Production That is Suppressed in Anergic But Exaggerated in Chronically BAFF-Exposed B Cells. J Immunol (2014) 192(8):3626–36. 10.4049/jimmunol.1302062 PMC3984038 24616478

[55] Jang KJ Mano H Aoki K Hayashi T Muto A Nambu Y. Mitochondrial Function Provides Instructive Signals for Activation-Induced B-cell Fates. Nat Commun (2015) 6:6750. 10.1038/ncomms7750 25857523 PMC4403446

[56] Setoguchi K Misaki Y Terauchi Y Yamauchi T Kawahata K Kadowaki T. Peroxisome Proliferator-Activated Receptor-Gamma Haploinsufficiency Enhances B Cell Proliferative Responses and Exacerbates Experimentally Induced Arthritis. J Clin Invest (2001) 108(11):1667–75. 10.1172/JCI13202 PMC200985 11733562

[57] Wright HL Lyon M Chapman EA Moots RJ Edwards SW. Rheumatoid Arthritis Synovial Fluid Neutrophils Drive Inflammation Through Production of Chemokines, Reactive Oxygen Species, and Neutrophil Extracellular Traps. Front Immunol (2021) 11:584116. 10.3389/fimmu.2020.584116 33469455 PMC7813679

[58] Injarabian L Devin A Ransac S Marteyn BS. Neutrophil Metabolic Shift During Their Lifecycle: Impact on Their Survival and Activation. Int J Mol Sci (2019) 21(1):287. 10.3390/ijms21010287 PMC6981538 31906243

[59] Caielli S Athale S Domic B Murat E Chandra M Banchereau R. Oxidized Mitochondrial Nucleoids Released by Neutrophils Drive Type I Interferon Production in Human Lupus. J Exp Med (2016) 213(5):697–713. 10.1084/jem.20151876 27091841 PMC4854735

[60] Contis A Mitrovic S Lavie J Douchet I Lazaro E Truchetet ME. Neutrophil-Derived Mitochondrial DNA Promotes Receptor Activator of Nuclear Factor Kappa B and its Ligand Signalling in Rheumatoid Arthritis. Rheumatology (2017) 56(7):1200–5. 10.1093/rheumatology/kex041 28340056

[61] Lood C Blanco LP Purmalek MM Carmona-Rivera C De Ravin SS Smith CK. Neutrophil Extracellular Traps Enriched in Oxidized Mitochondrial DNA are Interferogenic and Contribute to Lupus-Like Disease. Nat Med (2016) 22(2):146–53. 10.1038/nm.4027 PMC4742415 26779811

[62] Biniecka M Kennedy A Fearon U Ng CT Veale DJ O’Sullivan JN. Oxidative Damage in Synovial Tissue is Associated With In Vivo Hypoxic Status in the Arthritic Joint. Ann Rheum Dis (2010) 69(6):1172–8. 10.1136/ard.2009.111211 19706618

[63] Biniecka M Fox E Gao W Ng CT Veale DJ Fearon U. Hypoxia Induces Mitochondrial Mutagenesis and Dysfunction in Inflammatory Arthritis. Arthritis Rheum (2011) 63(8):2172–82. 10.1002/art.30395 21484771

[64] Ng CT Biniecka M Kennedy A McCormick J Fitzgerald O Bresnihan B. Synovial Tissue Hypoxia and Inflammation In Vivo. Ann Rheum Dis (2010) 69(7):1389–95. 10.1136/ard.2009.119776 PMC2946116 20439288

[65] Deng W Feng XB Li X Wang DD Sun LY. Hypoxia-Inducible Factor 1 in Autoimmune Diseases. Cell Immunol (2016) 303:7–15. 10.1016/j.cellimm.2016.04.001 27071377

[66] McGarry T Biniecka M Veale DJ Fearon U. Hypoxia, Oxidative Stress and Inflammation. Free Radical Biol Med (2018) 125:15–24. 10.1016/j.freeradbiomed.2018.03.042 29601945

[67] Semenza GL. Oxygen-Dependent Regulation of Mitochondrial Respiration by Hypoxia-Inducible Factor 1. Biochem J (2007) 405(1):1–9. 10.1042/BJ20070389 17555402

[68] Fukuda R Zhang HF Kim JW Shimoda L Dang CV Semenza GL. HIF-1 Regulates Cytochrome Oxidase Subunits to Optimize Efficiency of Respiration in Hypoxic Cells. Cell (2007) 129(1):111–22. 10.1016/j.cell.2007.01.047 17418790

[69] Papandreou I Cairns RA Fontana L Lim AL Denko NC. HIF-1 Mediates Adaptation to Hypoxia by Actively Downregulating Mitochondrial Oxygen Consumption. Cell Metab (2006) 3(3):187–97. 10.1016/j.cmet.2006.01.012 16517406

[70] Wang D Wang Q Yan G Qiao Y Zhu B Liu B. Hypoxia Induces Lactate Secretion and Glycolytic Efflux by Downregulating Mitochondrial Pyruvate Carrier Levels in Human Umbilical Vein Endothelial Cells. Mol Med Rep (2018) 18(2):1710–7. 10.3892/mmr.2018.9079 29845198

[71] Fuhrmann DC Olesch C Kurrle N Schnütgen F Zukunft S Fleming I. Chronic Hypoxia Enhances β-Oxidation-Dependent Electron Transport Via Electron Transferring Flavoproteins. Cells (2019) 8(2):172. 10.3390/cells8020172 PMC6406996 30781698

[72] Morten KJ Badder L Knowles HJ. Differential Regulation of HIF-mediated Pathways Increases Mitochondrial Metabolism and ATP Production in Hypoxic Osteoclasts. J Pathol (2013) 229(5):755–64. 10.1002/path.4159 PMC3618370 23303559

[73] Poderoso JJ Helfenberger K Poderoso C. The Effect of Nitric Oxide on Mitochondrial Respiration. Nitric Oxid Biol Chem (2019) 88:61–72. 10.1016/j.niox.2019.04.005 30999001

[74] Clementi E Brown GC Feelisch M Moncada S. Persistent Inhibition of Cell Respiration by Nitric Oxide: Crucial Role of S-nitrosylation of Mitochondrial Complex I and Protective Action of Glutathione. Proc Natl Acad Sci USA (1998) 95(13):7631–6. 10.1073/pnas.95.13.7631 PMC22706 9636201

[75] Palmieri EM Gonzalez-Cotto M Baseler WA Davies LC Ghesquière B Maio N. Nitric Oxide Orchestrates Metabolic Rewiring in M1 Macrophages by Targeting Aconitase 2 and Pyruvate Dehydrogenase. Nat Commun (2020) 11(1):698. 10.1038/s41467-020-14433-7 32019928 PMC7000728

[76] Bailey JD Diotallevi M Nicol T McNeill E Shaw A Chuaiphichai S. Nitric Oxide Modulates Metabolic Remodeling in Inflammatory Macrophages Through TCA Cycle Regulation and Itaconate Accumulation. Cell Rep (2019) 28(1):218–+. 10.1016/j.celrep.2019.06.018 PMC6616861 31269442

[77] Everts B Amiel E van der Windt GJW Freitas TC Chott R Yarasheski KE. Commitment to Glycolysis Sustains Survival of NO-producing Inflammatory Dendritic Cells. Blood (2012) 120(7):1422–31. 10.1182/blood-2012-03-419747 PMC3423780 22786879

[78] Van den Bossche J Baardman J Otto NA van der Velden S Neele AE van den Berg SM. Mitochondrial Dysfunction Prevents Repolarization of Inflammatory Macrophages. Cell Rep (2016) 17(3):684–96. 10.1016/j.celrep.2016.09.008 27732846

[79] Bogdan C. Nitric Oxide Synthase in Innate and Adaptive Immunity: An Update. Trends Immunol (2015) 36(3):161–78. 10.1016/j.it.2015.01.003 25687683

[80] Gross TJ Kremens K Powers LS Brink B Knutson T Domann FE. Epigenetic Silencing of the Human Nos2 Gene: Rethinking the Role of Nitric Oxide in Human Macrophage Inflammatory Responses. J Immunol (2014) 192(5):2326–38. 10.4049/jimmunol.1301758 PMC3943971 24477906

[81] Thomas AC Mattila JT. “Of Mice and Men”: Arginine Metabolism in Macrophages. Front Immunol (2014) 5:479. 10.3389/fimmu.2014.00479 25339954 PMC4188127

[82] StClair EW Wilkinson WE Lang T Sanders L Misukonis MA Gilkeson GS. Increased Expression of Blood Mononuclear Cell Nitric Oxide Synthase Type 2 in Rheumatoid Arthritis Patients. J Exp Med (1996) 184(3):1173–8. 10.1084/jem.184.3.1173 PMC2192765 9064335

[83] Nagy G Barcza M Gonchoroff N Phillips PE Perl A. Nitric Oxide-Dependent Mitochondrial Biogenesis Generates Ca2+ Signaling Profile of Lupus T Cells. J Immunol (2004) 173(6):3676–83. 10.4049/jimmunol.173.6.3676 PMC4034140 15356113

[84] Nagy G Clark JM Buzas E Gorman C Pasztoi M Koncz A. Nitric Oxide Production of T Lymphocytes is Increased in Rheumatoid Arthritis. Immunol Lett (2008) 118(1):55–8. 10.1016/j.imlet.2008.02.009 18396335

[85] Divakaruni AS Hsieh WY Minarrieta L Duong TN Kim KKO Desousa BR. Etomoxir Inhibits Macrophage Polarization by Disrupting Coa Homeostasis. Cell Metab (2018) 28(3):490–+. 10.1016/j.cmet.2018.06.001 PMC6125190 30043752

[86] O’Connor RS Guo LL Ghassemi S Snyder NW Worth AJ Weng L. The CPT1a Inhibitor, Etomoxir Induces Severe Oxidative Stress At Commonly Used Concentrations. Sci Rep (2018) 8:6289. 10.1038/s41598-018-24676-6 29674640 PMC5908836

[87] Namgaladze D Brune B. Fatty Acid Oxidation is Dispensable for Human Macrophage IL-4-induced Polarization. Biochim Et Biophys Acta Mol Cell Biol Lipids (2014) 1841(9):1329–35. 10.1016/j.bbalip.2014.06.007 24960101

[88] Rodgers LC Cole J Rattigan KM Barrett MP Kurian N McInnes IB. The Rheumatoid Synovial Environment Alters Fatty Acid Metabolism in Human Monocytes and Enhances CCL20 Secretion. Rheumatol (Oxford) (2020) 59(4):869–78. 10.1093/rheumatology/kez378 31497857

[89] Yang XY Zheng KD Lin K Zheng GF Zou H Wang JM. Energy Metabolism Disorder as a Contributing Factor of Rheumatoid Arthritis: A Comparative Proteomic and Metabolomic Study. PLoS One (2015) 10(7):15. 10.1371/journal.pone.0132695 PMC4492520 26147000

[90] Shen Y Wen Z Li Y Matteson EL Hong J Goronzy JJ. Metabolic Control of the Scaffold Protein TKS5 in Tissue-Invasive, Proinflammatory T Cells. Nat Immunol (2017) 18(9):1025–34. 10.1038/ni.3808 PMC5568495 28737753

[91] Zhou J Chen J Hu CF Xie ZJ Li HC Wei SS. Exploration of the Serum Metabolite Signature in Patients With Rheumatoid Arthritis Using Gas Chromatography-Mass Spectrometry. J Pharm Biomed Anal (2016) 127:60–7. 10.1016/j.jpba.2016.02.004 26879423

[92] Kim S Hwang J Xuan J Jung YH Cha HS Kim KH. Global Metabolite Profiling of Synovial Fluid for the Specific Diagnosis of Rheumatoid Arthritis From Other Inflammatory Arthritis. PLoS One (2014) 9(6):9. 10.1371/journal.pone.0097501 PMC4041724 24887281

[93] Frommer KW Schäffler A Rehart S Lehr A Müller-Ladner U Neumann E. Free Fatty Acids: Potential Proinflammatory Mediators in Rheumatic Diseases. Ann Rheum Dis (2015) 74(1):303–10. 10.1136/annrheumdis-2013-203755 24285492

[94] Kim JY Lim K Kim KH Kim JH Choi JS Shim SC. N-3 Polyunsaturated Fatty Acids Restore Th17 and Treg Balance in Collagen Antibody-Induced Arthritis. PLoS One (2018) 13(3):e0194331. 10.1371/journal.pone.0194331 29543869 PMC5854360

[95] Calder PC. Fatty Acids and Inflammation: The Cutting Edge Between Food and Pharma. Eur J Pharmacol (2011) 668 Suppl 1:S50–8. 10.1016/j.ejphar.2011.05.085 21816146

[96] Casanova-Vallve N Constantin-Teodosiu D Filer A Hardy RS Greenhaff PL Chapman V. Skeletal Muscle Dysregulation in Rheumatoid Arthritis: Metabolic and Molecular Markers in a Rodent Model and Patients. PLoS One (2020) 15(7):e0235702. 10.1371/journal.pone.0235702 32634159 PMC7340297

[97] Kieler M Hofmann M Schabbauer G. More Than Just Protein Building Blocks: How Amino Acids and Related Metabolic Pathways Fuel Macrophage Polarization. FEBS J (2021) 21:15715. 10.1111/febs.15715 PMC8359336 33460504

[98] Wise DR Thompson CB. Glutamine Addiction: A New Therapeutic Target in Cancer. Trends Biochem Sci (2010) 35(8):427–33. 10.1016/j.tibs.2010.05.003 PMC2917518 20570523

[99] Tannahill GM Curtis AM Adamik J Palsson-McDermott EM McGettrick AF Goel G. Succinate is an Inflammatory Signal That Induces IL-1 Beta Through HIF-1 Alpha. Nature (2013) 496(7444):238–+. 10.1038/nature11986 PMC4031686 23535595

[100] Arts RJW Novakovic B ter Horst R Carvalho A Bekkering S Lachmandas E. Glutaminolysis and Fumarate Accumulation Integrate Immunometabolic and Epigenetic Programs in Trained Immunity. Cell Metab (2016) 24(6):807–19. 10.1016/j.cmet.2016.10.008 PMC5742541 27866838

[101] Johnson MO Wolf MM Madden MZ Andrejeva G Sugiura A Contreras DC. Distinct Regulation of Th17 and Th1 Cell Differentiation by Glutaminase-Dependent Metabolism. Cell (2018) 175(7):1780–+. 10.1016/j.cell.2018.10.001 PMC6361668 30392958

[102] Liu PS Wang HP Li XY Chao T Christen TTS Christen S. Alpha-Ketoglutarate Orchestrates Macrophage Activation Through Metabolic and Epigenetic Reprogramming. Nat Immunol (2017) 18(9):985–+. 10.1038/ni.3796 28714978

[103] Xu T Stewart KM Wang XH Liu K Xie M Ryu JK. Metabolic Control of T(H)17 and Induced T-reg Cell Balance by an Epigenetic Mechanism. Nature (2017) 548(7666):228–+. 10.1038/nature23475 PMC6701955 28783731

[104] Jha AK Huang SCC Sergushichev A Lampropoulou V Ivanova Y Loginicheva E. Network Integration of Parallel Metabolic and Transcriptional Data Reveals Metabolic Modules That Regulate Macrophage Polarization. Immunity (2015) 42(3):419–30. 10.1016/j.immuni.2015.02.005 25786174

[105] Takahashi S Saegusa J Sendo S Okano T Akashi K Irino Y. Glutaminase 1 Plays a Key Role in the Cell Growth of Fibroblast-Like Synoviocytes in Rheumatoid Arthritis. Arthritis Res Ther (2017) 19(1):76. 10.1186/s13075-017-1283-3 28399896 PMC5387190

[106] Klysz D Tai XG Robert PA Craveiro M Cretenet G Oburoglu L. Glutamine-Dependent Alpha-Ketoglutarate Production Regulates the Balance Between T Helper 1 Cell and Regulatory T Cell Generation. Sci Signaling (2015) 8(396):12. 10.1126/scisignal.aab2610 26420908

[107] Kono M Yoshida N Maeda K Tsokos GC. Transcriptional Factor ICER Promotes Glutaminolysis and the Generation of Th17 Cells. Proc Natl Acad Sci USA (2018) 115(10):2478–83. 10.1073/pnas.1714717115 PMC5877961 29463741

[108] Ueda Y Saegusa J Okano T Sendo S Yamada H Nishimura K. Additive Effects of Inhibiting Both mTOR and Glutamine Metabolism on the Arthritis in SKG Mice. Sci Rep (2019) 9:11. 10.1038/s41598-019-42932-1 31011190 PMC6476881

[109] Kapoor SR Filer A Fitzpatrick MA Fisher BA Taylor PC Buckley CD. Metabolic Profiling Predicts Response to Anti-Tumor Necrosis Factor Alpha Therapy in Patients With Rheumatoid Arthritis. Arthritis Rheum (2013) 65(6):1448–56. 10.1002/art.37921 PMC3715109 23460124

[110] Priori R Casadei L Valerio M Scrivo R Valesini G Manetti C. H-1-Nmr-Based Metabolomic Study for Identifying Serum Profiles Associated With the Response to Etanercept in Patients With Rheumatoid Arthritis. PLoS One (2015) 10(11):14. 10.1371/journal.pone.0138537 PMC4641599 26558759

[111] Littlewood-Evans A Sarret S Apfel V Loesle P Dawson J Zhang J. GPR91 Senses Extracellular Succinate Released From Inflammatory Macrophages and Exacerbates Rheumatoid Arthritis. J Exp Med (2016) 213(9):1655–62. 10.1084/jem.20160061 PMC4995082 27481132

[112] Hügle T Kovacs H Heijnen IA Daikeler T Baisch U Hicks JM. Synovial Fluid Metabolomics in Different Forms of Arthritis Assessed by Nuclear Magnetic Resonance Spectroscopy. Clin Exp Rheumatol (2012) 30(2):240–5. 22410098

[113] Lee YJ Mun S Lee YR Lee S Kwon S Kim D. A Discovery of Screening Markers for Rheumatoid Arthritis by Liquid Chromatography Mass Spectrometry: A Metabolomic Approach. Int J Rheum Dis (2020) 23(10):1353–62. 10.1111/1756-185X.13935 32845094

[114] Young SP Kapoor SR Viant MR Byrne JJ Filer A Buckley CD. The Impact of Inflammation on Metabolomic Profiles in Patients With Arthritis. Arthritis Rheum (2013) 65(8):2015–23. 10.1002/art.38021 PMC3840700 23740368

[115] Coras R Murillo-Saich JD Guma M. Circulating Pro- and Anti-Inflammatory Metabolites and Its Potential Role in Rheumatoid Arthritis Pathogenesis. Cells (2020) 9(4):827. 10.3390/cells9040827 PMC7226773 32235564

[116] Cordes T Wallace M Michelucci A Divakaruni AS Sapcariu SC Sousa C. Immunoresponsive Gene 1 and Itaconate Inhibit Succinate Dehydrogenase to Modulate Intracellular Succinate Levels. J Biol Chem (2016) 291(27):14274–84. 10.1074/jbc.M115.685792 PMC4933182 27189937

[117] Mills E O’Neill LA. Succinate: A Metabolic Signal in Inflammation. Trends Cell Biol (2014) 24(5):313–20. 10.1016/j.tcb.2013.11.008 24361092

[118] Li Y Liu Y Wang C Xia WR Zheng JY Yang J. Succinate Induces Synovial Angiogenesis in Rheumatoid Arthritis Through Metabolic Remodeling and HIF-1 Alpha/VEGF Axis. Free Radical Biol Med (2018) 126:1–14. 10.1016/j.freeradbiomed.2018.07.009 30030103

[119] Cummins EP Berra E Comerford KM Ginouves A Fitzgerald KT Seeballuck F. Prolyl Hydroxylase-1 Negatively Regulates IkappaB Kinase-Beta, Giving Insight Into Hypoxia-Induced NFkappaB Activity. Proc Natl Acad Sci USA (2006) 103(48):18154–9. 10.1073/pnas.0602235103 PMC1643842 17114296

[120] Rubic T Lametschwandtner G Jost S Hinteregger S Kund J Carballido-Perrig N. Triggering the Succinate Receptor GPR91 on Dendritic Cells Enhances Immunity. Nat Immunol (2008) 9(11):1261–9. 10.1038/ni.1657 18820681

[121] Saraiva AL Veras FP Peres RS Talbot J de Lima KA Luiz JP. Succinate Receptor Deficiency Attenuates Arthritis by Reducing Dendritic Cell Traffic and Expansion of T(h)17 Cells in the Lymph Nodes. FASEB J (2018) 32(12):6550–8. 10.1096/fj.201800285 29894669

[122] Leblond A Allanore Y Avouac J. Targeting Synovial Neoangiogenesis in Rheumatoid Arthritis. Autoimmun Rev (2017) 16(6):594–601. 10.1016/j.autrev.2017.04.005 28414154

[123] Biniecka M Canavan M McGarry T Gao W McCormick J Cregan S. Dysregulated Bioenergetics: A Key Regulator of Joint Inflammation. Ann Rheum Dis (2016) 75(12):2192–200. 10.1136/annrheumdis-2015-208476 PMC5136702 27013493

[124] Harber KJ de Goede KE Verberk SGS Meinster E de Vries HE van Weeghel M. Succinate Is an Inflammation-Induced Immunoregulatory Metabolite in Macrophages. Metabolites (2020) 10(9):14. 10.3390/metabo10090372 PMC7569821 32942769

[125] Keiran N Ceperuelo-Mallafré V Calvo E Hernández-Alvarez MI Ejarque M Núñez-Roa C. SUCNR1 Controls an Anti-Inflammatory Program in Macrophages to Regulate the Metabolic Response to Obesity. Nat Immunol (2019) 20(5):581–92. 10.1038/s41590-019-0372-7 30962591

[126] Wu JY Huang TW Hsieh YT Wang YF Yen CC Lee GL. Cancer-Derived Succinate Promotes Macrophage Polarization and Cancer Metastasis Via Succinate Receptor. Mol Cell (2020) 77(2):213–27.e5. 10.1016/j.molcel.2019.10.023 31735641

[127] Zhunussova A Sen B Friedman L Tuleukhanov S Brooks AD Sensenig R. Tumor Microenvironment Promotes Dicarboxylic Acid Carrier-Mediated Transport of Succinate to Fuel Prostate Cancer Mitochondria. Am J Cancer Res (2015) 5(5):1665–79. PMC4497434 26175936

[128] Reddy A Bozi LHM Yaghi OK Mills EL Xiao HP Nicholson HE. Ph-Gated Succinate Secretion Regulates Muscle Remodeling in Response to Exercise. Cell (2020) 183(1):62–+. 10.1016/j.cell.2020.08.039 PMC7778787 32946811

[129] Cummings NA Nordby GL. Nordby: MEASUREMENT of SYNOVIAL Fluid PH in NORMAL and ARTHRITIC Knees. Arthritis Rheum (1966) 9(1):47–. 10.1002/art.1780090106 4952418

[130] Certo M Marone G de Paulis A Mauro C Pucino V. Lactate: Fueling the Fire Starter. Wiley Interdiscip Rev Sys Biol Med (2020) 12(3):15. 10.1002/wsbm.1474 PMC7187281 31840439

[131] Neveu MA Beziere N Daniels R Bouzin C Comment A Schwenck J. Lactate Production Precedes Inflammatory Cell Recruitment in Arthritic Ankles: An Imaging Study. Mol Imaging Biol (2020) 22(5):1324–32. 10.1007/s11307-020-01510-y PMC7497460 32514887

[132] O’Neill LAJ Artyomov MN. Itaconate: The Poster Child of Metabolic Reprogramming in Macrophage Function. Nat Rev Immunol (2019) 19(5):273–81. 10.1038/s41577-019-0128-5 30705422

[133] Lampropoulou V Sergushichev A Bambouskova M Nair S Vincent EE Loginicheva E. Itaconate Links Inhibition of Succinate Dehydrogenase With Macrophage Metabolic Remodeling and Regulation of Inflammation. Cell Metab (2016) 24(1):158–66. 10.1016/j.cmet.2016.06.004 PMC5108454 27374498

[134] Li YK Zhang P Wang CC Han CF Meng J Liu XG. Immune Responsive Gene 1 (Irg1) Promotes Endotoxin Tolerance by Increasing A20 Expression in Macrophages Through Reactive Oxygen Species. J Biol Chem (2013) 288(23):16225–34. 10.1074/jbc.M113.454538 PMC3675562 23609450

[135] Mills EL Ryan DG Prag HA Dikovskaya D Menon D Zaslona Z. Itaconate is an Anti-Inflammatory Metabolite That Activates Nrf2 Via Alkylation of KEAP1. Nature (2018) 556(7699):113–+. 10.1038/nature25986 PMC6047741 29590092

[136] Li C Chen B Fang Z Leng YF Wang DW Chen FQ. Metabolomics in the Development and Progression of Rheumatoid Arthritis: A Systematic Review. Joint Bone Spine (2020) 87(5):425–30. 10.1016/j.jbspin.2020.05.005 32473419

[137] Daly R Blackburn G Best C Goodyear CS Mudaliar M Burgess K. Changes in Plasma Itaconate Elevation in Early Rheumatoid Arthritis Patients Elucidates Disease Activity Associated Macrophage Activation. Metabolites (2020) 10(6):12. 10.3390/metabo10060241 PMC7344783 32531990

[138] Michopoulos F Karagianni N Whalley NM Firth MA Nikolaou C Wilson ID. Targeted Metabolic Profiling of the Tg197 Mouse Model Reveals Itaconic Acid as a Marker of Rheumatoid Arthritis. J Proteome Res (2016) 15(12):4579–90. 10.1021/acs.jproteome.6b00654 27704840

[139] Li A van Luijk N ter Beek M Caspers M Punt P van der Werf M. A Clone-Based Transcriptomics Approach for the Identification of Genes Relevant for Itaconic Acid Production in Aspergillus. Fungal Genet Biol (2011) 48(6):602–11. 10.1016/j.fgb.2011.01.013 21324422

[140] Li R Zhang P Wang Y Tao K. Itaconate: A Metabolite Regulates Inflammation Response and Oxidative Stress. Oxid Med Cell Longev (2020) 2020:5404780. 10.1155/2020/5404780 32724492 PMC7382747

[141] Puchalska P Huang X Martin SE Han X Patti GJ Crawford PA. Isotope Tracing Untargeted Metabolomics Reveals Macrophage Polarization-State-Specific Metabolic Coordination Across Intracellular Compartments. iScience (2018) 9:298–313. 10.1016/j.isci.2018.10.029 30448730 PMC6240706

[142] Wang L Hauenstein AV. The NLRP3 Inflammasome: Mechanism of Action, Role in Disease and Therapies. Mol Asp Med (2020) 76:100889. 10.1016/j.mam.2020.100889 32859386

[143] Moossavi M Parsamanesh N Bahrami A Atkin SL Sahebkar A. Role of the NLRP3 Inflammasome in Cancer. Mol Cancer (2018) 17(1):158. 10.1186/s12943-018-0900-3 30447690 PMC6240225

[144] Spel L Martinon F. Inflammasomes Contributing to Inflammation in Arthritis. Immunolog Rev (2020) 294(1):48–62. 10.1111/imr.12839 31944344

[145] Swanson KV Deng M Ting JPY. The NLRP3 Inflammasome: Molecular Activation and Regulation to Therapeutics. Nat Rev Immunol (2019) 19(8):477–89. 10.1038/s41577-019-0165-0 PMC7807242 31036962

[146] Wu KK Cheung SW Cheng KK. Nlrp3 Inflammasome Activation in Adipose Tissues and Its Implications on Metabolic Diseases. Int J Mol Sci (2020) 21(11):4184. 10.3390/ijms21114184 PMC7312293 32545355

[147] Kolbrink B Riebeling T Kunzendorf U Krautwald S. Plasma Membrane Pores Drive Inflammatory Cell Death. Front Cell Dev Biol (2020) 8:817. 10.3389/fcell.2020.00817 32974349 PMC7471660

[148] Hughes MM O’Neill LAJ. Metabolic Regulation of NLRP3. Immunolog Rev (2018) 281(1):88–98. 10.1111/imr.12608 29247992

[149] Bauernfeind F Bartok E Rieger A Franchi L Núñez G Hornung V. Cutting Edge: Reactive Oxygen Species Inhibitors Block Priming, But Not Activation, of the NLRP3 Inflammasome. J Immunol (2011) 187(2):613–7. 10.4049/jimmunol.1100613 PMC3131480 21677136

[150] Meissner F Seger RA Moshous D Fischer A Reichenbach J Zychlinsky A. Inflammasome Activation in NADPH Oxidase Defective Mononuclear Phagocytes From Patients With Chronic Granulomatous Disease. Blood (2010) 116(9):1570–3. 10.1182/blood-2010-01-264218 PMC2938844 20495074

[151] Zhou R Yazdi AS Menu P Tschopp J. A Role for Mitochondria in NLRP3 Inflammasome Activation. Nature (2011) 469(7329):221–5. 10.1038/nature09663 21124315

[152] Liu YB Fiskum G Schubert D. Generation of Reactive Oxygen Species by the Mitochondrial Electron Transport Chain. J Neurochem (2002) 80(5):780–7. 10.1046/j.0022-3042.2002.00744.x 11948241

[153] Liu XT Zhang X Ding Y Zhou W Tao L Lu P. Nuclear Factor E2-Related Factor-2 Negatively Regulates Nlrp3 Inflammasome Activity by Inhibiting Reactive Oxygen Species-Induced NLRP3 Priming. Antioxid Redox Signaling (2017) 26(1):28–43. 10.1089/ars.2015.6615 PMC5198158 27308893

[154] Nakahira K Haspel JA Rathinam VAK Lee SJ Dolinay T Lam HC. Autophagy Proteins Regulate Innate Immune Responses by Inhibiting the Release of Mitochondrial DNA Mediated by the NALP3 Inflammasome. Nat Immunol (2011) 12(3):222–U57. 10.1038/ni.1980 21151103 PMC3079381

[155] Biasizzo M Kopitar-Jerala N. Interplay Between Nlrp3 Inflammasome and Autophagy. Front Immunol (2020) 11:591803. 10.3389/fimmu.2020.591803 33163006 PMC7583715

[156] Rahman T Nagar A Duffy EB Okuda K Silverman N Harton JA. Nlrp3 Sensing of Diverse Inflammatory Stimuli Requires Distinct Structural Features. Front Immunol (2020) 11:1828. 10.3389/fimmu.2020.01828 32983094 PMC7479093

[157] Sadatomi D Nakashioya K Mamiya S Honda S Kameyama Y Yamamura Y. Mitochondrial Function is Required for Extracellular ATP-induced NLRP3 Inflammasome Activation. J Biochem (2017) 161(6):503–12. 10.1093/jb/mvw098 28096454

[158] Moon JS Lee S Park MA Siempos II Haslip M Lee PJ. UCP2-Induced Fatty Acid Synthase Promotes NLRP3 Inflammasome Activation During Sepsis. J Clin Invest (2015) 125(2):665–80. 10.1172/jci78253 PMC4319445 25574840

[159] Moon JS Nakahira K Chung KP DeNicola GM Koo MJ Pabón MA. NOX4-Dependent Fatty Acid Oxidation Promotes NLRP3 Inflammasome Activation in Macrophages. Nat Med (2016) 22(9):1002–12. 10.1038/nm.4153 PMC5204248 27455510

[160] Subramanian N Natarajan K Clatworthy MR Wang Z Germain RN. The Adaptor Mavs Promotes Nlrp3 Mitochondrial Localization and Inflammasome Activation. Cell (2013) 153(2):348–61. 10.1016/j.cell.2013.02.054 PMC3632354 23582325

[161] Elliott EI Miller AN Banoth B Iyer SS Stotland A Weiss JP. Cutting Edge: Mitochondrial Assembly of the NLRP3 Inflammasome Complex is Initiated At Priming. J Immunol (2018) 200(9):3047–52. 10.4049/jimmunol.1701723 PMC5916517 29602772

[162] Iyer SS He Q Janczy JR Elliott EI Zhong Z Olivier AK. Mitochondrial Cardiolipin is Required for Nlrp3 Inflammasome Activation. Immunity (2013) 39(2):311–23. 10.1016/j.immuni.2013.08.001 PMC3779285 23954133

[163] Park S Juliana C Hong S Datta P Hwang I Fernandes-Alnemri T. The Mitochondrial Antiviral Protein Mavs Associates With NLRP3 and Regulates its Inflammasome Activity. J Immunol (2013) 191(8):4358–66. 10.4049/jimmunol.1301170 PMC3848201 24048902

[164] Shimada K Crother TR Karlin J Dagvadorj J Chiba N Chen S. Oxidized Mitochondrial Dna Activates the NLRP3 Inflammasome During Apoptosis. Immunity (2012) 36(3):401–14. 10.1016/j.immuni.2012.01.009 PMC3312986 22342844

[165] Dayer JM. The Pivotal Role of Interleukin-1 in the Clinical Manifestations of Rheumatoid Arthritis. Rheumatology (2003) 42:3–10. 10.1093/rheumatology/keg326 12817089

[166] Guo C Fu R Wang S Huang Y Li X Zhou M. NLRP3 Inflammasome Activation Contributes to the Pathogenesis of Rheumatoid Arthritis. Clin Exp Immunol (2018) 194(2):231–43. 10.1111/cei.13167 PMC6194337 30277570

[167] Wu Y He X Huang N Yu J Shao B. A20: A Master Regulator of Arthritis. Arthritis Res Ther (2020) 22(1):220. 10.1186/s13075-020-02281-1 32958016 PMC7504854

[168] Vande Walle L Van Opdenbosch N Jacques P Fossoul A Verheugen E Vogel P. Negative Regulation of the NLRP3 Inflammasome by A20 Protects Against Arthritis. Nature (2014) 512(7512):69–+. 10.1038/nature13322 PMC4126806 25043000

[169] Cohen SB Moreland LW Cush JJ Greenwald MW Block S Shergy WJ. A Multicentre, Double Blind, Randomised, Placebo Controlled Trial of Anakinra (Kineret), a Recombinant Interleukin 1 Receptor Antagonist, in Patients With Rheumatoid Arthritis Treated With Background Methotrexate. Ann Rheum Dis (2004) 63(9):1062–8. 10.1136/ard.2003.016014 PMC1755108 15082469

[170] Mertens M Singh JA. Anakinra for Rheumatoid Arthritis: A Systematic Review. J Rheumatol (2009) 36(6):1118–25. 10.3899/jrheum.090074 19447938

[171] Gracie JA Forsey RJ Chan WL Gilmour A Leung BP Greer MR. A Proinflammatory Role for IL-18 in Rheumatoid Arthritis. J Clin Invest (1999) 104(10):1393–401. 10.1172/jci7317 PMC409841 10562301

[172] Rooney T Murphy E Benito M Roux-Lombard P FitzGerald O Dayer JM. Synovial Tissue interleukin-18 Expression and the Response to Treatment in Patients With Inflammatory Arthritis. Ann Rheum Dis (2004) 63(11):1393–8. 10.1136/ard.2003.016428 PMC1754818 15479888

[173] Plater-Zyberk C Joosten LAB Helsen MMA Sattonnet-Roche P Siegfried C Alouani S. Therapeutic Effect of Neutralizing Endogenous IL-18 Activity in the Collagen-Induced Model of Arthritis. J Clin Invest (2001) 108(12):1825–32. 10.1172/jci200112097 PMC209462 11748266

[174] Wei XQ Leung BP Arthur HML McInnes IB Liew FY. Reduced Incidence and Severity of Collagen-Induced Arthritis in Mice Lacking IL-18. J Immunol (2001) 166(1):517–21. 10.4049/jimmunol.166.1.517 11123331

[175] Krysko DV Agostinis P Krysko O Garg AD Bachert C Lambrecht BN. Emerging Role of Damage-Associated Molecular Patterns Derived From Mitochondria in Inflammation. Trends Immunol (2011) 32(4):157–64. 10.1016/j.it.2011.01.005 21334975

[176] da Silva JLG Passos DF Bernardes VM Leal DBR. ATP and Adenosine: Role in the Immunopathogenesis of Rheumatoid Arthritis. Immunol Lett (2019) 214:55–64. 10.1016/j.imlet.2019.08.009 31479688

[177] Elliott MR Chekeni FB Trampont PC Lazarowski ER Kadl A Walk SF. Nucleotides Released by Apoptotic Cells Act as a Find-Me Signal to Promote Phagocytic Clearance. Nature (2009) 461(7261):282–U165. 10.1038/nature08296 19741708 PMC2851546

[178] Loredo GA Benton HP. ATP and UTP Activate Calcium-Mobilizing P-2U-like Receptors and Act Synergistically With Interleukin-1 to Stimulate Prostaglandin E-2 Release From Human Rheumatoid Synovial Cells. Arthritis Rheum (1998) 41(2):246–55. 10.1002/1529-0131(199802)41:2<246::aid-art8>3.3.co;2-9 9485082

[179] Cao F Hu LQ Yao SR Hu Y Wang DG Fan YG. P2X7 Receptor: A Potential Therapeutic Target for Autoimmune Diseases. Autoimmun Rev (2019) 18(8):767–77. 10.1016/j.autrev.2019.06.009 31181327

[180] Labasi JM Petrushova N Donovan C McCurdy S Lira P Payette MM. Absence of the P2X(7) Receptor Alters Leukocyte Function and Attenuates an Inflammatory Response. J Immunol (2002) 168(12):6436–45. 10.4049/jimmunol.168.12.6436 12055263

[181] Fan ZD Zhang YY Guo YH Huang N Ma HH Huang H. Involvement of P2X7 Receptor Signaling on Regulating the Differentiation of Th17 Cells and Type II Collagen-Induced Arthritis in Mice. Sci Rep (2016) 6:12. 10.1038/srep35804 27775097 PMC5075966

[182] Portales-Cervantes L Nino-Moreno P Doniz-Padilla L Baranda-Candido L Garcia-Hernandez M Salgado-Bustamante M. Expression and Function of the P2X(7) Purinergic Receptor in Patients With Systemic Lupus Erythematosus and Rheumatoid Arthritis. Hum Immunol (2010) 71(8):818–25. 10.1016/j.humimm.2010.05.008 20493226

[183] Al-Shukaili A Al-Kaabi J Hassan B. A Comparative Study of Interleukin-1 Beta Production and P2x(7) Expression After Atp Stimulation by Peripheral Blood Mononuclear Cells Isolated From Rheumatoid Arthritis Patients and Normal Healthy Controls. Inflammation (2008) 31(2):84–90. 10.1007/s10753-007-9052-0 18040764

[184] Li MX Yang CY Wang YH Song W Jia LN Peng XX. The Expression of P2X7 Receptor on Th1, Th17, and Regulatory T Cells in Patients With Systemic Lupus Erythematosus or Rheumatoid Arthritis and Its Correlations With Active Disease. J Immunol (2020) 205(7):1752–+. 10.4049/jimmunol.2000222 32868411

[185] Ghiringhelli F Apetoh L Tesniere A Aymeric L Ma YT Ortiz C. Activation of the NLRP3 Inflammasome in Dendritic Cells Induces IL-1 Beta-Dependent Adaptive Immunity Against Tumors. Nat Med (2009) 15(10):1170–U99. 10.1038/nm.2028 19767732

[186] Eleftheriadis T Pissas G Liakopoulos V Stefanidis I. Cytochrome C as a Potentially Clinical Useful Marker of Mitochondrial and Cellular Damage. Front Immunol (2016) 7:279. 10.3389/fimmu.2016.00279 27489552 PMC4951490

[187] Pullerits R Bokarewa M Jonsson IM Verdrengh M Tarkowski A. Extracellular Cytochrome C, a Mitochondrial Apoptosis-Related Protein, Induces Arthritis. Rheumatology (2005) 44(1):32–9. 10.1093/rheumatology/keh406 15367748

[188] Mamula MJ Jemmerson R Hardin JA. The SPECIFICITY of HUMAN Anti-Cytochrome-C AUTOANTIBODIES That ARISE in Autoimmune-Disease. J Immunol (1990) 144(5):1835–40. 1689756

[189] West AP Shadel GS. Mitochondrial DNA in Innate Immune Responses and Inflammatory Pathology. Nat Rev Immunol (2017) 17(6):363–75. 10.1038/nri.2017.21 PMC7289178 28393922

[190] Hajizadeh S DeGroot J TeKoppele JM Tarkowski A Collins LV. Extracellular Mitochondrial DNA and Oxidatively Damaged DNA in Synovial Fluid of Patients With Rheumatoid Arthritis. Arthritis Res Ther (2003) 5(5):R234–40. 10.1186/ar787 PMC193725 12932286

[191] Nakayama H Otsu K. Mitochondrial DNA as an Inflammatory Mediator in Cardiovascular Diseases. Biochem J (2018) 475:839–52. 10.1042/bcj20170714 PMC5840331 29511093

[192] Collins LV Hajizadeh S Holme E Jonsson IM Tarkowski A. Endogenously Oxidized Mitochondrial DNA Induces In Vivo and In Vitro Inflammatory Responses. J Leukoc Biol (2004) 75(6):995–1000. 10.1189/jlb.0703328 14982943

[193] Rykova E Sizikov A Roggenbuck D Antonenko O Bryzgalov L Morozkin E. Circulating DNA in Rheumatoid Arthritis: Pathological Changes and Association With Clinically Used Serological Markers. Arthritis Res Ther (2017) 19:10. 10.1186/s13075-017-1295-z 28464939 PMC5414163

[194] Fearon U Canavan M Biniecka M Veale DJ. Hypoxia, Mitochondrial Dysfunction and Synovial Invasiveness in Rheumatoid Arthritis. Nat Rev Rheumatol (2016) 12(7):385–97. 10.1038/nrrheum.2016.69 27225300

[195] Berthelot JM Le Goff B Neel A Maugars Y Hamidou M. Netosis: At the Crossroads of Rheumatoid Arthritis, Lupus, and Vasculitis. Joint Bone Spine (2017) 84(3):255–62. 10.1016/j.jbspin.2016.05.013 27426444

[196] Khandpur R Carmona-Rivera C Vivekanandan-Giri A Gizinski A Yalavarthi S Knight JS. Nets Are a Source of Citrullinated Autoantigens and Stimulate Inflammatory Responses in Rheumatoid Arthritis. Sci Trans Med (2013) 5(178):10. 10.1126/scitranslmed.3005580 PMC3727661 23536012

[197] Spengler J Lugonja B Ytterberg AJ Zubarev RA Creese AJ Pearson MJ. Release of Active Peptidyl Arginine Deiminases by Neutrophils can Explain Production of Extracellular Citrullinated Autoantigens in Rheumatoid Arthritis Synovial Fluid. Arthritis Rheumatol (2015) 67(12):3135–45. 10.1002/art.39313 PMC4832324 26245941

[198] Vorobjeva N Galkin I Pletjushkina O Golyshev S Zinovkin R Prikhodko A. Mitochondrial Permeability Transition Pore is Involved in Oxidative Burst and NETosis of Human Neutrophils. Biochim Et Biophys Acta Mol Basis Dis (2020) 1866(5):15. 10.1016/j.bbadis.2020.165664 31926265

[199] Grishko VI Ho R Wilson GL Pearsall AW. Diminished Mitochondrial DNA Integrity and Repair Capacity in OA Chondrocytes. Osteoarthr Cartil (2009) 17(1):107–13. 10.1016/j.joca.2008.05.009 PMC3640312 18562218

[200] Kim J Xu M Xo R Mates A Wilson GL Pearsall AW. Mitochondrial DNA Damage is Involved in Apoptosis Caused by Pro-Inflammatory Cytokines in Human OA Chondrocytes. Osteoarthr Cartil (2010) 18(3):424–32. 10.1016/j.joca.2009.09.008 19822235

[201] Pizzuto M Pelegrin P. Cardiolipin in Immune Signaling and Cell Death. Trends Cell Biol (2020) 30(11):892–903. 10.1016/j.tcb.2020.09.004 33011017

[202] Marziale A Bettacchioli E Picart G Nafai S Galinat H Meroni PL. Antiphospholipid Autoantibody Detection is Important in All Patients With Systemic Autoimmune Diseases. J Autoimmun (2020) 115:102524. 10.1016/j.jaut.2020.102524 32693965

[203] Kao W Gu R Jia Y Wei X Fan H Harris J. A Formyl Peptide Receptor Agonist Suppresses Inflammation and Bone Damage in Arthritis. Br J Pharmacol (2014) 171(17):4087–96. 10.1111/bph.12768 PMC4243981 24824742

[204] Chen LN Duvvuri B Grigull J Jamnik R Wither JE Wu GE. Experimental Evidence That Mutated-Self Peptides Derived From Mitochondrial DNA Somatic Mutations Have the Potential to Trigger Autoimmunity. Hum Immunol (2014) 75(8):873–9. 10.1016/j.humimm.2014.06.012 24979674

[205] Da Sylva TR Connor A Mburu Y Keystone E Wu GE. Somatic Mutations in the Mitochondria of Rheumatoid Arthritis Synoviocytes. Arthritis Res Ther (2005) 7(4):R844–51. 10.1186/ar1752 PMC1175034 15987486

[206] Duvvuri B Duvvuri VR Wang C Chen LN Wagar LE Jamnik V. The Human Immune System Recognizes Neopeptides Derived From Mitochondrial Dna Deletions. J Immunol (2014) 192(10):4581–91. 10.4049/jimmunol.1300774 24733843

[207] Harty LC Biniecka M O’Sullivan J Fox E Mulhall K Veale DJ. Mitochondrial Mutagenesis Correlates With the Local Inflammatory Environment in Arthritis. Ann Rheum Dis (2012) 71(4):582–8. 10.1136/annrheumdis-2011-200245 22121133

[208] Du J Yu S Wang D Chen S Zheng Y Wang N. Germline and Somatic mtDNA Mutation Spectrum of Rheumatoid Arthritis Patients in the Taizhou Area, China. Rheumatol (Oxford) (2020) 59(10):2982–91. 10.1093/rheumatology/keaa063 32159782

[209] Mitsunaga S Hosomichi K Okudaira Y Nakaoka H Suzuki Y Kuwana M. Aggregation of Rare/Low-Frequency Variants of the Mitochondria Respiratory Chain-Related Proteins in Rheumatoid Arthritis Patients. J Hum Genet (2015) 60(8):449–54. 10.1038/jhg.2015.50 26016412

[210] Murphy MP Hartley RC. Mitochondria as a Therapeutic Target for Common Pathologies. Nat Rev Drug Discovery (2018) 17(12):865–86. 10.1038/nrd.2018.174 30393373

[211] Piranavan P Bhamra M Perl A. Metabolic Targets for Treatment of Autoimmune Diseases. Immunometabolism (2020) 2(2):e200012. 10.20900/immunometab20200012 32341806 PMC7184931

[212] Bedoui Y Guillot X Sélambarom J Guiraud P Giry C Jaffar-Bandjee MC. Methotrexate an Old Drug With New Tricks. Int J Mol Sci (2019) 20(20):5023. 10.3390/ijms20205023 PMC6834162 31658782

[213] Herman S Zurgil N Deutsch M. Low Dose Methotrexate Induces Apoptosis With Reactive Oxygen Species Involvement in T Lymphocytic Cell Lines to a Greater Extent Than in Monocytic Lines. Inflammation Res (2005) 54(7):273–80. 10.1007/s00011-005-1355-8 16134056

[214] Paul M Hemshekhar M Thushara RM Sundaram MS NaveenKumar SK Naveen S. Methotrexate Promotes Platelet Apoptosis Via JNK-Mediated Mitochondrial Damage: Alleviation by N-Acetylcysteine and N-Acetylcysteine Amide. PLoS One (2015) 10(6):e0127558. 10.1371/journal.pone.0127558 26083398 PMC4471342

[215] Huang C Hsu P Hung Y Liao Y Liu C Hour C. Ornithine Decarboxylase Prevents Methotrexate-Induced Apoptosis by Reducing Intracellular Reactive Oxygen Species Production. Apoptosis (2005) 10(4):895–907. 10.1007/s10495-005-2947-z 16133879

[216] Lee SY Park SH Lee SW Lee SH Son MK Choi YH. Synoviocyte Apoptosis may Differentiate Responder and non-Responder Patients to Methotrexate Treatment in Rheumatoid Arthritis. Arch Pharm Res (2014) 37(10):1286–94. 10.1007/s12272-014-0365-x 24988987

[217] Al Maruf A O’Brien PJ Naserzadeh P Fathian R Salimi A Pourahmad J. Methotrexate Induced Mitochondrial Injury and Cytochrome C Release in Rat Liver Hepatocytes. Drug Chem Toxicol (2018) 41(1):51–61. 10.1080/01480545.2017.1289221 28298149

[218] Heidari R Ahmadi A Mohammadi H Ommati MM Azarpira N Niknahad H. Mitochondrial Dysfunction and Oxidative Stress are Involved in the Mechanism of Methotrexate-Induced Renal Injury and Electrolytes Imbalance. BioMed Pharmacother (2018) 107:834–40. 10.1016/j.biopha.2018.08.050 30142545

[219] Kolli VK Natarajan K Isaac B Selvakumar D Abraham P. Mitochondrial Dysfunction and Respiratory Chain Defects in a Rodent Model of Methotrexate-Induced Enteritis. Hum Exp Toxicol (2014) 33(10):1051–65. 10.1177/0960327113515503 24347301

[220] Papadopoli DJ Ma EH Roy D Russo M Bridon G Avizonis D. Methotrexate Elicits Pro-Respiratory and Anti-Growth Effects by Promoting AMPK Signaling. Sci Rep (2020) 10(1):7838. 10.1038/s41598-020-64460-z 32398698 PMC7217946

[221] Schultz M Keeling SO Katz SJ Maksymowych WP Eurich DT Hall JJ. Clinical Effectiveness and Safety of Leflunomide in Inflammatory Arthritis: A Report From the RAPPORT Database With Supporting Patient Survey. Clin Rheumatol (2017) 36(7):1471–8. 10.1007/s10067-017-3687-5 28550389

[222] Xuan JK Ren Z Qing T Couch L Shi LM Tolleson WH. Mitochondrial Dysfunction Induced by Leflunomide and its Active Metabolite. Toxicology (2018) 396:33–45. 10.1016/j.tox.2018.02.003 29427785 PMC5909954

[223] Miret-Casals L Sebastian D Brea J Rico-Leo EM Palacin M Fernandez-Salguero PM. Identification of New Activators of Mitochondrial Fusion Reveals a Link Between Mitochondrial Morphology and Pyrimidine Metabolism. Cell Chem Biol (2018) 25(3):268–+. 10.1016/j.chembiol.2017.12.001 29290623

[224] Yu MF Nguyen ND Huang YQ Lin D Fujimoto TN Molkentine JM. Mitochondrial Fusion Exploits a Therapeutic Vulnerability of Pancreatic Cancer. JCI Insight (2019) 4(16):16. 10.1172/jci.insight.126915 PMC6777817 31335325

[225] Klotz L Eschborn M Lindner M Liebmann M Herold M Janoschka C. Teriflunomide Treatment for Multiple Sclerosis Modulates T Cell Mitochondrial Respiration With Affinity-Dependent Effects. Sci Trans Med (2019) 11(490):17. 10.1126/scitranslmed.aao5563 31043571

[226] Fang J Uchiumi T Yagi M Matsumoto S Amamoto R Takazaki S. Dihydro-Orotate Dehydrogenase is Physically Associated With the Respiratory Complex and its Loss Leads to Mitochondrial Dysfunction. Biosci Rep (2013) 33(2):e00021. 10.1042/BSR20120097 23216091 PMC3564035

[227] Gadangi P Longaker M Naime D Levin RI Recht PA Montesinos MC. The Anti-Inflammatory Mechanism of Sulfasalazine is Related to Adenosine Release At Inflamed Sites. J Immunol (1996) 156(5):1937–41. 8596047

[228] Liptay S Fulda S Schanbacher M Bourteele S Ferri KF Kroemer G. Molecular Mechanisms of Sulfasalazine-Induced T-cell Apoptosis. Br J Pharmacol (2002) 137(5):608–20. 10.1038/sj.bjp.0704870 PMC1573529 12381674

[229] Xie Y Hou W Song X Yu Y Huang J Sun X. Ferroptosis: Process and Function. Cell Death Differ (2016) 23(3):369–79. 10.1038/cdd.2015.158 PMC5072448 26794443

[230] Gout PW Buckley AR Simms CR Bruchovsky N. Sulfasalazine, a Potent Suppressor of Lymphoma Growth by Inhibition of the X(C)(-) Cystine Transporter: A New Action for an Old Drug. Leukemia (2001) 15(10):1633–40. 10.1038/sj.leu.2402238 11587223

[231] Yu HC Yang CC Jian L Guo SP Chen R Li K. Sulfasalazine-Induced Ferroptosis in Breast Cancer Cells is Reduced by the Inhibitory Effect of Estrogen Receptor on the Transferrin Receptor. Oncol Rep (2019) 42(2):826–38. 10.3892/or.2019.7189 31173262

[232] Niknahad H Heidari R Mohammadzadeh R Ommati MM Khodaei F Azarpira N. Sulfasalazine Induces Mitochondrial Dysfunction and Renal Injury. Renal Fail (2017) 39(1):745–53. 10.1080/0886022x.2017.1399908 PMC6446160 29214868

[233] Biniecka M Kennedy A Ng CT Chang TC Balogh E Fox E. Successful Tumour Necrosis Factor (TNF) Blocking Therapy Suppresses Oxidative Stress and Hypoxia-Induced Mitochondrial Mutagenesis in Inflammatory Arthritis. Arthritis Res Ther (2011) 13(4):9. 10.1186/ar3424 PMC3239359 21787418

[234] Costa NT Iriyoda TMV Alfieri DF Simao ANC Dichi I. Influence of Disease-Modifying Antirheumatic Drugs on Oxidative and Nitrosative Stress in Patients With Rheumatoid Arthritis. Inflammopharmacology (2018) 26(5):1151–64. 10.1007/s10787-018-0514-9 30062629

[235] Meugnier E Coury F Tebib J Ferraro-Peyret C Rome S Bienvenu J. Gene Expression Profiling in Peripheral Blood Cells of Patients With Rheumatoid Arthritis in Response to anti-TNF-alpha Treatments. Physiol Genomics (2011) 43(7):365–71. 10.1152/physiolgenomics.00127.2010 21266503

[236] Derambure C Dzangue-Tchoupou G Berard C Vergne N Hiron M D’Agostino MA. Pre-Silencing of Genes Involved in the Electron Transport Chain (etc) Pathway is Associated With Responsiveness to Abatacept in Rheumatoid Arthritis. Arthritis Res Ther (2017) 19:13. 10.1186/s13075-017-1319-8 28545499 PMC5445375

[237] Jamilloux Y El Jammal T Vuitton L Gerfaud-Valentin M Kerever S Seve P. JAK Inhibitors for the Treatment of Autoimmune and Inflammatory Diseases. Autoimmun Rev (2019) 18(11):14. 10.1016/j.autrev.2019.102390 31520803

[238] McGarry T Orr C Wade S Biniecka M Gallagher L Low C. Jak/Stat Blockade Alters Synovial Bioenergetics, Mitochondrial Function, and Proinflammatory Mediators in Rheumatoid Arthritis. Arthritis Rheumatol (2018) 70(12):1959–70. 10.1002/art.40569 29790294

[239] Moisan A Lee YK Zhang JD Hudak CS Meyer CA Prummer M. White-to-Brown Metabolic Conversion of Human Adipocytes by JAK Inhibition. Nat Cell Biol (2015) 17(1):57–+. 10.1038/ncb3075 PMC4276482 25487280

[240] Melville AR Kearsley-Fleet L Buch MH Hyrich KL. Understanding Refractory Rheumatoid Arthritis: Implications for a Therapeutic Approach. Drugs (2020) 80(9):849–57. 10.1007/s40265-020-01309-9 32361822

[241] Nagy G van Vollenhoven RF. Sustained Biologic-Free and Drug-Free Remission in Rheumatoid Arthritis, Where are We Now? Arthritis Res Ther (2015) 17:181. 10.1186/s13075-015-0707-1 26235544 PMC4522973

[242] Huang N Perl A. Metabolism as a Target for Modulation in Autoimmune Diseases. Trends Immunol (2018) 39(7):562–76. 10.1016/j.it.2018.04.006 29739666

[243] Perl A. Activation of mTOR (Mechanistic Target of Rapamycin) in Rheumatic Diseases. Nat Rev Rheumatol (2016) 12(3):169–82. 10.1038/nrrheum.2015.172 PMC5314913 26698023

[244] Salvatore T Pafundi PC Galiero R Gjeloshi K Masini F Acierno C. Metformin: A Potential Therapeutic Tool for Rheumatologists. Pharm (Basel) (2020) 13(9):234. 10.3390/ph13090234 PMC7560003 32899806

[245] Kang KY Kim YK Yi H Kim J Jung HR Kim IJ. Metformin Downregulates Th17 Cells Differentiation and Attenuates Murine Autoimmune Arthritis. Int Immunopharmacol (2013) 16(1):85–92. 10.1016/j.intimp.2013.03.020 23557965

[246] Son HJ Lee J Lee SY Kim EK Park MJ Kim KW. Metformin Attenuates Experimental Autoimmune Arthritis Through Reciprocal Regulation of Th17/Treg Balance and Osteoclastogenesis. Mediators Inflammation (2014) 2014:973986. 10.1155/2014/973986 PMC4158168 25214721

[247] Yan H Zhou HF Hu Y Pham CT. Suppression of Experimental Arthritis Through AMP-activated Protein Kinase Activation and Autophagy Modulation. J Rheum Dis Treat (2015) 1(1):5. 10.23937/2469-5726/1510005 26120598 PMC4479345

[248] Hou X Song J Li XN Zhang L Wang X Chen L. Metformin Reduces Intracellular Reactive Oxygen Species Levels by Upregulating Expression of the Antioxidant Thioredoxin Via the AMPK-FOXO3 Pathway. Biochem Biophys Res Commun (2010) 396(2):199–205. 10.1016/j.bbrc.2010.04.017 20398632

[249] Sato N Takasaka N Yoshida M Tsubouchi K Minagawa S Araya J. Metformin Attenuates Lung Fibrosis Development Via NOX4 Suppression. Respir Res (2016) 17(1):107. 10.1186/s12931-016-0420-x 27576730 PMC5006432

[250] Andreev-Andrievskiy AA Kolosova NG Stefanova NA Lovat MV Egorov MV Manskikh VN. Efficacy of Mitochondrial Antioxidant Plastoquinonyl-decyl-triphenylphosphonium Bromide (SkQ1) in the Rat Model of Autoimmune Arthritis. Oxid Med Cell Longev (2016) 2016:8703645. 10.1155/2016/8703645 27293517 PMC4887630

[251] Cetinkaya A Bulbuloglu E Kurutas EB Kantarceken B. N-Acetylcysteine Ameliorates Methotrexate-Induced Oxidative Liver Damage in Rats. Med Sci Monit (2006) 12(8):BR274–8. 16865059

[252] Jhun J Lee S Kim SY Na HS Kim EK Kim JK. Combination Therapy With Metformin and Coenzyme Q10 in Murine Experimental Autoimmune Arthritis. Immunopharmacol Immunotoxicol (2016) 38(2):103–12. 10.3109/08923973.2015.1122619 26681425

[253] McInnes IB Cruwys S Bowers K Braddock M. Targeting the P2X(7) Receptor in Rheumatoid Arthritis: Biological Rationale for P2X(7) Antagonism. Clin Exp Rheumatol (2014) 32(6):878–82. 25288220

[254] Keystone EC Wang MM Layton M Hollis S McInnes IB Team DCS. Clinical Evaluation of the Efficacy of the P2X(7) Purinergic Receptor Antagonist AZD9056 on the Signs and Symptoms of Rheumatoid Arthritis in Patients With Active Disease Despite Treatment With Methotrexate or Sulphasalazine. Ann Rheum Dis (2012) 71(10):1630–5. 10.1136/annrheumdis-2011-143578 22966146

[255] Stock TC Bloom BJ Wei N Ishaq S Park W Wang X. Efficacy and Safety of CE-224,535, an Antagonist of P2X(7) Receptor, in Treatment of Patients With Rheumatoid Arthritis Inadequately Controlled by Methotrexate. J Rheumatol (2012) 39(4):720–7. 10.3899/jrheum.110874 22382341

[256] Zhang F Wei K Slowikowski K Fonseka CY Rao DA Kelly S. Defining Inflammatory Cell States in Rheumatoid Arthritis Joint Synovial Tissues by Integrating Single-Cell Transcriptomics and Mass Cytometry. Nat Immunol (2019) 20(7):928–+. 10.1038/s41590-019-0378-1 PMC6602051 31061532

[257] Berridge MV McConnell MJ Grasso C Bajzikova M Kovarova J Neuzil J. Horizontal Transfer of Mitochondria Between Mammalian Cells: Beyond Co-Culture Approaches. Curr Opin Genet Dev (2016) 38:75–82. 10.1016/j.gde.2016.04.003 27219870

[258] Levoux J Prola A Lafuste P Gervais M Chevallier N Koumaiha Z. Platelets Facilitate the Wound-Healing Capability of Mesenchymal Stem Cells by Mitochondrial Transfer and Metabolic Reprogramming. Cell Metab (2020) 33(2):283–99.e9. 10.1016/j.cmet.2020.12.006 33400911

[259] Fuhrmann DC Brune B. Mitochondrial Composition and Function Under the Control of Hypoxia. Redox Biol (2017) 12:208–15. 10.1016/j.redox.2017.02.012 PMC5333533 28259101

[260] Garaude J. Reprogramming of Mitochondrial Metabolism by Innate Immunity. Curr Opin Immunol (2019) 56:17–23. 10.1016/j.coi.2018.09.010 30286442

[261] Lapuente-Brun E Moreno-Loshuertos R Acin-Perez R Latorre-Pellicer A Colas C Balsa E. Supercomplex Assembly Determines Electron Flux in the Mitochondrial Electron Transport Chain. Science (2013) 340(6140):1567–70. 10.1126/science.1230381 23812712

